# METTL1-mediated m^7^G methylation maintains pluripotency in human stem cells and limits mesoderm differentiation and vascular development

**DOI:** 10.1186/s13287-020-01814-4

**Published:** 2020-07-22

**Authors:** Yujie Deng, Zhongyang Zhou, Weidong Ji, Shuibin Lin, Min Wang

**Affiliations:** 1 Center for Translational Medicine, The First Affiliated Hospital, Sun Yat-sen University, Guangzhou, 510080 China; 2 Department of Rehabilitation Medicine, The Sixth Affiliated Hospital, Sun Yat-sen University, Guangzhou, 510000 China

**Keywords:** N7-methylguanosine (m^7^G), Human induced pluripotent stem cells (hiPSCs), Pluripotency, Differentiation, Mesoderm, Vasculogenesis

## Abstract

**Background:**

7-Methylguanosine (m^7^G) is one of the most conserved modifications in nucleosides within tRNAs and rRNAs. It plays essential roles in the regulation of mRNA export, splicing, and translation. Recent studies highlighted the importance of METTL1-mediated m^7^G tRNA methylome in the self-renewal of mouse embryonic stem cells (mESCs) through its ability to regulate mRNA translation. However, the exact mechanisms by which METTL1 regulates pluripotency and differentiation in human induced pluripotent stem cells (hiPSCs) remain unknown. In this study, we evaluated the functions and underlying molecular mechanisms of METTL1 in regulating hiPSC self-renewal and differentiation in vivo and in vitro.

**Methods:**

By establishing METTL1 knockdown (KD) hiPSCs, gene expression profiling was performed by RNA sequencing followed by pathway analyses. Anti-m^7^G northwestern assay was used to identify m^7^G modifications in tRNAs and mRNAs. Polysome profiling was used to assess the translation efficiency of the major pluripotent transcription factors. Moreover, the in vitro and in vivo differentiation capacities of METTL1-KD hiPSCs were assessed in embryoid body (EB) formation and teratoma formation assays.

**Results:**

METTL1 silencing resulted in alterations in the global m^7^G profile in hiPSCs and reduced the translational efficiency of stem cell marker genes. METTL1-KD hiPSCs exhibited reduced pluripotency with slower cell cycling. Moreover, METTL1 silencing accelerates hiPSC differentiation into EBs and promotes the expression of mesoderm-related genes. Similarly, METTL1 knockdown enhances teratoma formation and mesoderm differentiation in vivo by promoting cell proliferation and angiogenesis in nude mice.

**Conclusion:**

Our findings provided novel insight into the critical role of METTL1-mediated m^7^G modification in the regulation of hiPSC pluripotency and differentiation, as well as its potential roles in vascular development and the treatment of vascular diseases.

## Introduction

Somatic cells can be transformed into induced pluripotent stem cells (iPSCs) functionally equivalent to embryonic stem cells (ESCs) by simultaneously introducing four transcription factors (TFs) Oct4, Sox2, Klf4, and c-Myc [1–3]. The critical role of these factors in maintaining pluripotency is highlighted by the fact that cell differentiation is enhanced when these pluripotent factors are suppressed [4, 5]. Recent studies have led to the identification of additional TFs and microRNAs (miRNAs) that can also affect the pluripotency and self-renewal capacity of iPSCs [6, 7].

Now, several studies have indicated that epigenetic modifications regulate stem cell fate decision and development [8–10]. One of the hotspots in epigenetic regulation, N6-methyladenosine (m^6^A) mRNA methylation, has been observed during embryogenesis and is believed to be involved in the regulation of stem cell development [11]. However, the role of post-transcriptional modifications of RNA, including 7-methylguanosine (m^7^G), in the regulation of hiPSCs fate remains unclear. METTL1 is an m^7^G RNA methyltransferase, and mutations in the human m^7^G methyltransferase complex METTL1/WDR4 can lead to primordial dwarfism and brain malformation [12]. Our previous study found that METTL1-mediated m^7^G tRNA modification regulates mRNA translation and the self-renewal, pluripotency, and neural lineage differentiation in mouse embryonic stem cells (mESCs) [13]. However, the differentiation of hiPSC is accompanied by the translational regulation of multiple genes [14, 15]. Thus, we hypothesize that METTL1 may also play an important role in hiPSC pluripotency and differentiation.

In this study, we investigated whether METTL1-mediated m^7^G methylation regulates pluripotency of hiPSCs. We find that METTL1 knockdown (KD) significantly decreased cell viability and changed the cell cycle of hiPSCs. Moreover, METTL1 KD significantly accelerates hiPSC differentiation toward the mesoderm fate and suppressed neuroectoderm differentiation in vitro. Most importantly, compared with control hiPSCs, METTL1-KD hiPSCs significantly enhance teratoma formation in vivo by promoting cell proliferation and angiogenesis in nude mice. Therefore, our study revealed that METTL1-mediated m^7^G modification regulates hiPSC pluripotency and differentiation, providing novel insights into vascular development and the treatment of vascular diseases.

## Materials and methods

### Chemical reagents and antibodies

The chemical reagents and antibodies used in this study are listed in the Supplementary Table S1 and Supplementary Table S2.

### Cell culture

HiPSCs and hESCs were maintained in matrigel-coated plates in mTeSR1 medium and under standard cell culture conditions. Cells were passaged every 4 to 5 days. 293T cells were maintained in DMEM supplemented with 10% FBS. All cells were maintained at 37 °C in a humidified 5% CO_2_ incubator. The detailed list of cell lines and mediums used for cell cultures was provided in the Supplementary Table S1 and Table S3.

### Knockdown of METTL1 in hiPSCs with shRNA lentivirus

The lentivirus expressing METTL1-targeting shRNAs has been previously described [13]. Briefly, 293T cells were transfected with the appropriate lentiviral vector using the Lipofectamine 3000 reagent (Invitrogen) according to the manufacturer’s instructions. The pLKO.1-shMETTL1-1 and pLKO.1-shMETTL1-5 vectors were used for METTL1 knockdown. The target and packaging plasmids TAT, HEPM2, RAII, and VSVG were used at a 4:1:1:1:1 ratio. Cells transduced with pLKO.1-shGFP viruses were used for control. Cell transduction was performed using polybrene. The transduced cells were selected with 1 μg/ml puromycin for 5 days.

### HiPSC self-renewal and proliferation analysis

To determine the proliferation capacity of hiPSCs, cells (2 × 10^4^) were seeded in 6-well plates, and the cell numbers were quantified on days 3, 5, and 7. For the colony formation assay, 500 cells were seeded in 6-well plates, followed by colony size and number quantification on day 7. Alkaline phosphatase staining was performed using an alkaline phosphatase detection kit (Merck Millipore, SCR004) following the manufacturer’s instructions. Briefly, 4–5 days after culturing cells on coverslips, cells were fixed with 4% paraformaldehyde for 1 min at room temperature. After washing with PBS, the cells were incubated with working buffer 15–45 min at room temperature and protected from light, followed by microscopy.

### RT-PCR and quantitative PCR analysis of gene expression

Total RNA was isolated using TRIzol, and cDNA synthesis was performed using ~ 600 ng of RNA and the all-in-one RT MasterMix kit (TransGen Biotech). For the qRT-PCR, the Step One Plus PCR system and SYBR Green Master Mix were used. All the relative gene expression was calculated using the function of comparative cycle threshold (Ct) and analyzed by 2^−ΔΔCt^ method. The primer sequences used in this study are listed in the Supplementary Table S4.

### Immunoprecipitation-Western blot

Cells were lysed in RIPA buffer containing protease inhibitors, and ~ 40 μg of protein was resolved by 10% SDS-PAGE. Proteins were then transferred onto PVDF membranes and blocked with 5% BSA in TBS-T. Membranes were probed overnight at 4 °C with primary antibodies against METTL1, FLAG, OCT4, SOX2, Nanog, CyclinD1, CyclinD2, CDK2, and ACTB. After washing, the membranes were incubated with the appropriate secondary antibody, and protein bands were visualized using the ECL system (Thermo Scientific).

### Determination of cell cycle analysis by flow cytometry

Cells were harvested using Accutase and fixed in ice-cold 70% ethanol overnight. Cells were stained with PI for 30 min, followed by flow cytometry analysis (Beckman Coulter, MoFlo Astrios EQ). Flow cytometry data were analyzed using Summit v. 5.3.

### Determination of m^7^G modification by Northwestern blot

Northwestern blot was performed to assess m^7^G modifications among METTL1-KD and control hiPSCs. RNA (~ 2 μg) was incubated in 2× TBE loading buffer at 95 °C for 5 min and then loaded onto 10% polyacrylamide-TBE urea gels. After electrophoresis, RNAs were transferred onto positively charged nylon membranes and UV-crosslinked. Membranes were probed with anti-m^7^G antibodies, and signal was detected using the ECL system.

### Determination of translation efficiency by sucrose-gradient centrifugation and polysome fractionation

Sucrose-gradient centrifugation and polysome fractionation were used to assess the impact of METTL1 silencing on the translation efficiency of stem cell markers. Cells (2 × 10^7^) were lysed in polysome cell extraction buffer on ice (the components are listed in the Supplementary Table S5), followed by centrifugation at 13,000×*g* for 15 min at 4 °C. Samples were loaded onto a 10–50% sucrose gradient and centrifuged at 36,000×*g* and 4 °C for 2.5 h. The polysome fraction was collected for qRT-PCR analysis. The relative translation efficiency was calculated by normalizing the mRNA levels in polysome fraction to the mRNA of gene expression.

### Induction of embryoid bodies (EBs)

To estimate the role of METTL1 in hiPSC differentiation in vitro, EB formation assay was performed as previously described [16, 17]. Briefly, hiPSCs were digested with Accutase, and 4 × 10^3^ cells/well were seeded in 96-well spheroid microplates (Corning, Cat #4515) in KO-DMEM medium supplemented with 10% KSR, 1% NEAA, 1 mM L-Glutamine, 50 μM 2-ME, and 10 μM Y-27632. Cells were incubated for 6 days, and the medium was refreshed daily.

### Induction of teratoma formation

To evaluate the role of METTL1 in hiPSC differentiation in vivo, animal experiments were performed in accordance with the guidelines provided by the First Affiliated Hospital of Sun Yat-sen University. Four-week-old BALB/C nude male mice were purchased from GemPharmatech Co. Ltd. (Nanjing, China). Mice were subcutaneously injected in the flank with 200 μl of PBS containing hiPSCs (6 × 10^6^ cells). At 6 weeks post-injection, teratomas were extracted from the nude mice for histological analyses.

### Determination of the expression of CD31 and SM22α by immunofluorescent staining

Tissue slides were fixed using 4% paraformaldehyde for 10 min and incubated with the rabbit anti-CD31 and mouse anti-SM22α antibodies overnight at 4 °C. Subsequently, sections were incubated with Alexa Fluor 488-conjugated donkey anti-rabbit IgG (1:400) or Alexa Fluor 594-conjugated donkey anti-mouse IgG (1:400) antibody for 1 h. Nuclei were counterstained with DAPI for 5 min. Coverslips were sealed and visualized under a confocal microscope (Zeiss).

### Determination of three layers and generation of teratomas by immunohistochemistry

Teratoma tissues were fixed in 4% paraformaldehyde at 4 °C overnight, and then embedded in paraffin and sectioned (7-μm thickness). Hematoxylin and eosin staining was performed on the sections. Tissue sections were also subjected to immunohistochemistry for Pan-CK, NeuN, vimentin, CD 31, and Ki67.

### Identification of METTL1-regulated genes by RNA sequencing (RNA-seq) analysis

A total of 2 μg RNA per sample was used for RNA sequencing (at least two replicates per sample). A complementary DNA library was prepared, and sequencing was performed by Beijing Annoroad Gene Technology Co. Ltd. After adaptor trimming and low-quality sequence filtering, the reads were mapped to the human reference genome version. Bowtie2 v2.2.3 was used for building the genome index, and sequences were then aligned to the reference genome using HISAT2 v2.1.0. Read counts were determined by HTSeq v0.6.0, and FPKM (fragments per kilobase million mapped reads) was then calculated to estimate the expression level of genes in each sample. The raw counts were then converted to reads per kilobase per million mapped reads (RPKM) using edegR. Genes with *q* ≤ 0.05 and |log2_ratio|≥ 1 were identified as differentially expressed genes (DEGs).

### Gene ontology (GO) and KEGG enrichment analysis

Functions of METTL1-regulated DEGs were analyzed by GO and KEGG in the Database for Annotation, Visualization, and Integrated Discovery (DAVID) (https://david.ncifcrf.gov/summary.jsp) [18] . GO enrichment analysis can predict the functional roles of DEGs associated with METTL1 knockdown in hiPSCs. GO enrichment in DEGs was assessed using the hypergeometric test, which uses the genes in the whole genome as background. GO terms with *q* < 0.05 were considered to be significantly enriched. KEGG analysis can define the pathways related to the DEGs associated with METTL1 knockdown. It was performed using the hypergeometric test, in which *P* values were adjusted for multiple comparisons, providing *q* values. KEGG terms with *q* < 0.05 were considered to be significantly enriched.

### Quantification and statistical analysis

All statistical analyses were performed using the GraphPad Prism 7.0 software (GraphPad Software, La Jolla, CA, USA). All results are representative of at least three independent experiments unless stated otherwise. The normality of the data was assessed using the Shapiro-Wilk test, while equality of group variance was assessed using the Brown-Forsythe test; these tests were performed using SigmaPlot 14 (Systat Software, San Jose, CA). Data are presented as the mean ± SEM of the biological replicates. Comparisons between two groups were performed by unpaired, two-tailed *t* test, while more than two groups were compared using one-way ANOVA followed by Bonferroni’s post hoc test or by two-way ANOVA. *P* values were two-tailed, and values < 0.05 were considered as statistically significant. *P < 0.05; **P < 0.01; ***P < 0.001.

## Results

### Gene expression profiling in METTL1 knockdown hiPSCs

To study the function of the tRNA m^7^G methyltransferase METTL1 in hiPSCs, METTL1 expression was silenced in hiPSCs using lentivirus expressing METTL1-targeting shRNAs (METTL1-KD). Pooled clones were selected, and knockdown efficiency of METTL1 was confirmed by qRT-PCR and western blot (Fig. S1a).

**Fig. 1:**
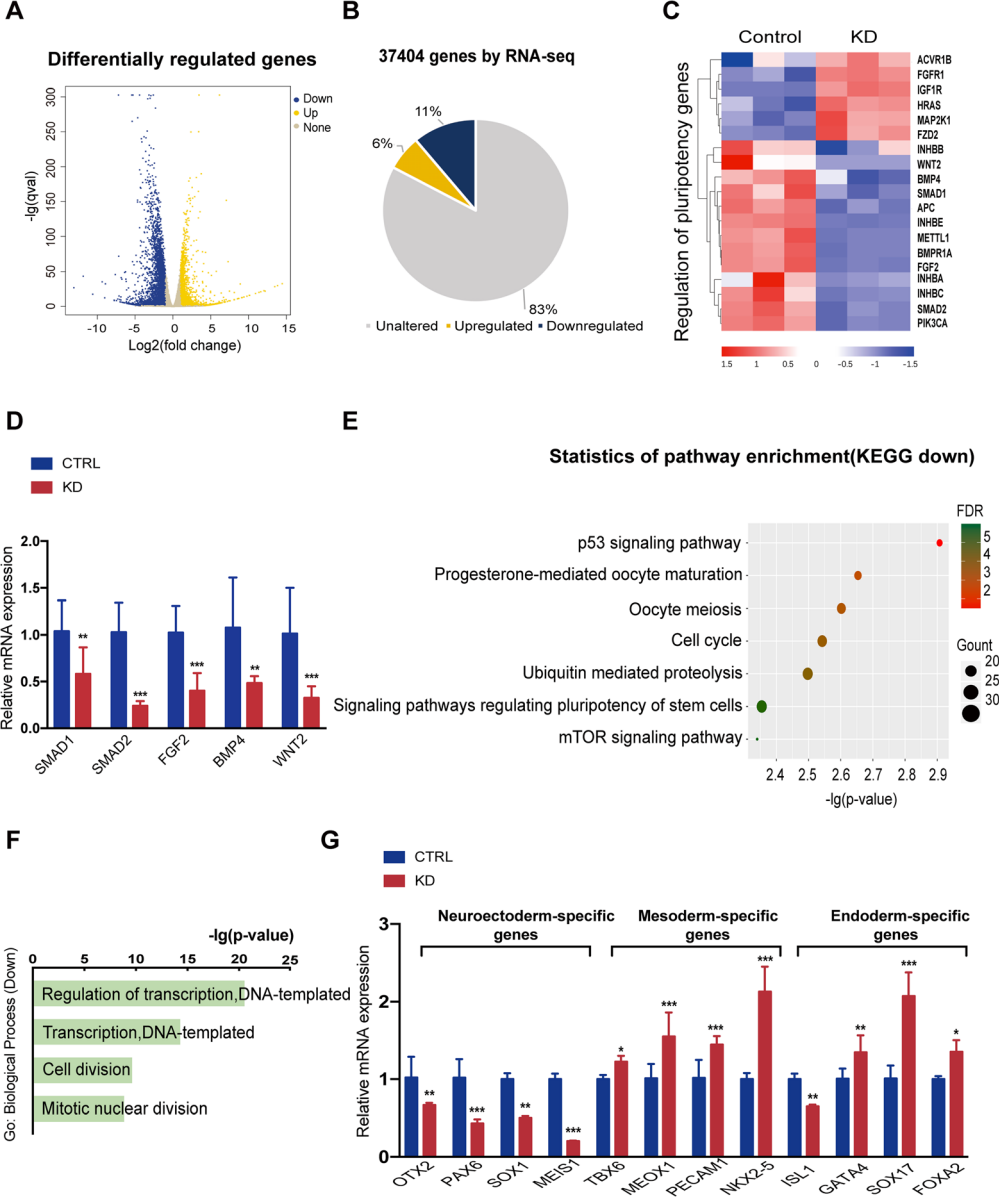
Identification of METTL1-regulated genes by RNA-seq analysis in hiPSCs. HiPSCs were transduced with shRNAs-METTL1, and pooled clones were selected. **a** Volcano plot illustrating differentially expressed genes (DEGs) between control and METTL1 knockdown (KD) hiPSCs. Genes upregulated and downregulated are shown in yellow and blue, respectively. Values are presented as the log2 of tag counts. **b** A total of 37,404 genes were differentially expressed, of which 2301 (6%) were upregulated and 4125 (11%) were downregulated in METTL1-KD hiPSCs. **c** Heatmap showing DEGs involved in stem cells pluripotency pathways. Each lane corresponds to an independent biological sample. Scale bar: log2 FPKM. **d** qRT-PCR analysis assessing the mRNA levels of genes involved in pluripotency, in control and METTL1-KD hiPSCs. **e** KEGG pathway analysis of genes downregulated in METTL1-KD hiPSCs. **f** Gene ontology (GO) functional clustering of genes downregulated in METTL1-KD hiPSCs; the top 4 most significant biological processes are shown. **g** qRT-PCR validation analysis shows the mRNA expression fold change of neuroectoderm-, endoderm- and mesoderm-specific genes in METTL1-KD vs. control hiPSCs. Data are presented as the mean ± SEM from three independent experiments. *P < 0.05; **P < 0.01; ***P < 0.001

RNA-seq analysis in hiPSCs led to the identification of 6426 DEGs, 2301 (6%) of which were upregulated, whilst 4125 (11%) were downregulated when METTL1 was silenced (Fig. 1a, b). Furthermore, KEGG pathway analysis showed that numerous pathways associated with stem cell regulation were significantly downregulated, including p53 signaling pathway, cell cycle, and the signaling pathways regulating stem cell pluripotency in METTL1-KD hiPSCs (Fig. 1e). Consistent with the pathway analysis, gene ontology (GO) enrichment analysis of the genes downregulated in METTL1-KD hiPSCs showed enrichment in regulation of transcription (DNA-templated) and cell division (Fig. 1f). Otherwise, in the upregulated pathways enriched by KEGG, we found that it is mainly related to immune responses, including systemic lupus erythematosus, graft-versus-host disease, and antigen processing presentation (Fig. S1c). qRT-PCR was performed to assess the expression of 5 representative genes differentially expressed following METTL1 silencing, which confirmed the reproducibility of our RNA-seq findings (Fig. 1c, d).

Additionally, a heatmap of downregulated genes revealed enrichment in neuroectoderm markers that are involved in neuronal cell development and differentiation (Fig. S1b). Consistent with the RNA-seq data, qRT-PCR verification showed that the expression of neuroectoderm-specific genes was downregulated in hiPSCs upon METTL1 silencing, whereas mesoderm- and endoderm-specific genes were expressed at higher levels (Fig. 1g). Of note, the downregulation of ISL1 is consistent with its expression pattern in endoderm and mesoderm/cardiogenic lineages as well as in neural stem cells [19, 20]. Taken together, these data suggested a role of METTL1 in maintaining hiPSC self-renewal and pluripotency.

### METTL1 promotes proliferation required for hiPSC self-renewal by regulating cell cycle

**Fig. 2:**
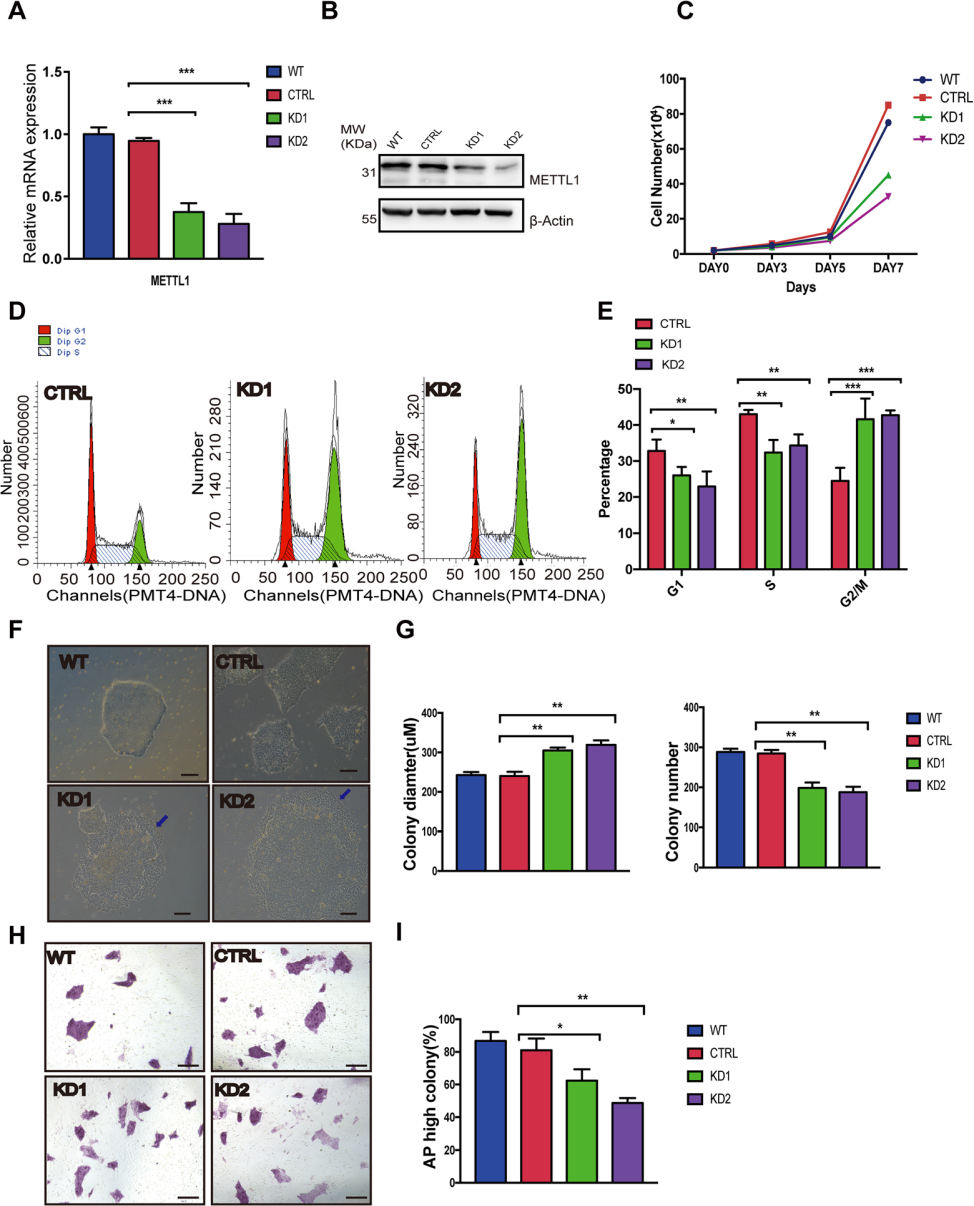
METTL1 is essential for hiPSC self-renewal and pluripotency. Different METTL1-KD hiPSC single-cell clones were selected, and two clones were used for further analyses. **a** qRT-PCR analysis of METTL1 mRNA expression in control and METTL1-KD hiPSC. **b** Western blot of the control and METTL1-KD samples with indicated antibodies. **c** Cell proliferation in control and METTL1-KD hiPSC. The cell numbers were quantified after 3, 5, and 7 days of culture; *n* = 3. **d**, **e** Cell cycle analysis in METTL1-KD and control hiPSC. **f**, **g** Colony formation assay of control and METTL1-KD hiPSC. Colony size and numbers were measured at day 7. **f** Representative images of the colonies. Scale bar, 100 μm. **g** Colony diameter and number quantification. **h**, **i** Alkaline phosphatase (AP) staining of control and METTL1-KD colonies. **h** Representative images; scale bar, 100 μm. **i** Percentage of AP positive colonies. The data are presented as mean ± SEM; *n* = 3; *P < 0.05; **P < 0.01; ***P < 0.001

We then assessed the relevance of METTL1-mediated m^7^G tRNA modifications in hiPSC proliferation and self- renewal using two different single-cell METTL1-KD hiPSC clones (Fig. 2a, b). We found that METTL1-KD cells proliferated slower compared with control hiPSCs (Fig. 2c). Moreover, cell cycle analysis revealed that the percentage of cells in the G2 phase was significantly higher in METTL1-KD hiPSCs compared with control cells (Fig. 2d, e). We also found that the mRNA and protein levels of critical cell cycle regulators were significantly altered in METTL1-KD hiPSCs (Fig. S2a, b).

Morphological analysis revealed that silencing of METTL1 resulted in flat colonies with increased surface area, as well as reduced the number of colonies (Fig. 2f, g). Notably, the cells found in the periphery of METTL1-KD hiPSC colonies appeared to be partially differentiated (blue arrows in Fig. 2f). A similar phenomenon was also observed in METTL1-KD hESCs (Fig. S2c), suggesting that METTL1 knockdown leads to a loss of self-renewal and promotes stem cell differentiation.

Since high levels of alkaline phosphatase (AP) can be used as a marker of undifferentiated state in stem cells [21], we sought to perform AP staining assays. We found a significant decrease in the AP-positive population in METTL1-KD hiPSCs (Fig. 2h, i). These data confirmed the critical role of METTL1 in maintaining stem cell self-renewal and preventing spontaneous differentiation.

### METTL1-mediated m^7^G methylome is required for hiPSC pluripotency by regulating the translation of stem cell markers

**Fig. 3:**
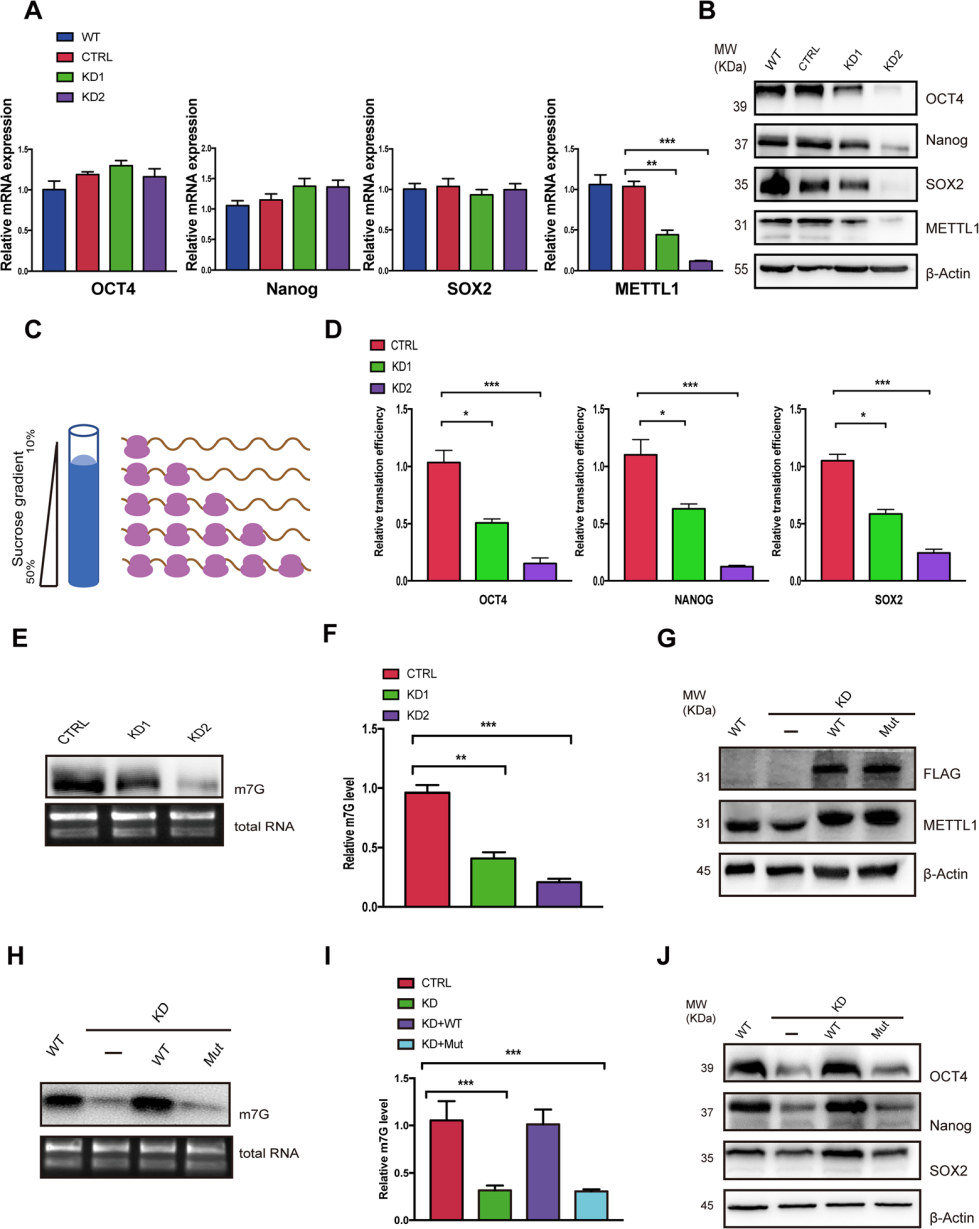
METTL1-mediated m^7^G methylation is required for OCT4, Nanog, and SOX2 translation in hiPSCs. **a**, **b** Effects of METTL1 KD on stem cell marker expression. **a** qRT-PCR analysis assessing the mRNA levels of OCT4, Nanog, and SOX2 in control and METTL1-KD hiPSCs. **b** Western blot showing OCT4, Nanog, and SOX2 protein levels in control and METTL1-KD hiPSCs. **c**, **d** Effects of METTL1-KD on translational efficiency of stem cell markers. **c** Schematic representation of the sucrose gradient procedure followed to isolate ribosome-free and ribosome-bound RNAs. **d** Total and polysome-fractionated RNAs from control and METTL1-KD cells were quantified by qRT-PCR, and translational efficiency was presented as relative percent of polysome associated mRNA to input mRNA of indicated stem cell markers. **e** Anti-m^7^G Northwestern blots (upper panel). RNAs were separated on TBE-urea gels, transferred to nylon membranes, and probed with anti-m^7^G antibodies. Expression was compared to total RNA controls (lower panel). **f** Quantification of relative m^7^G levels. **g**, **h** Rescue of stem cell markers expression by the exogenous expression of METTL1-WT but not an enzyme-inactive mutant (Mut). **g** Western blot analysis of reconstituted hiPSCs. **h** Anti-m^7^G Northwestern blot of m^7^G modifications (upper panel) and agarose gel electrophoresis of total RNA (lower panel) in METTL1-mutant samples. **i** Quantification of m^7^G levels. **j** Rescue of stem cell marker expression assessed by WB analysis. Data are presented as mean ± SEM; *n* = 3; **P < 0.01; ***P < 0.001

Since RNA-seq analysis indicated that signaling pathways regulating stem cell pluripotency were downregulated in METTL1-KD hiPSCs, the expression of pluripotency marker genes following METTL1 knockdown was investigated. We found that METTL1 downregulation resulted in only minor changes in the mRNA levels of stem cell markers Oct4, Nanog, and Sox2. However, protein levels of these markers were drastically decreased in METTL1-KD cells (Fig. 3a, b). Similar results were observed in two different hESC lines (Fig. S3a, b), suggesting that METTL1-mediated m^7^G RNA modifications might regulate the translation of stem cell markers.

We next sought to assess the impact of METTL1 silencing on the translation efficiency of stem cell markers. To this end, control and METTL1-KD hiPSCs were subjected to polysome fractionation via sucrose gradient centrifugation (Fig. 3c) and the mRNA levels of stem cell markers in each of the fractions were measured by qRT-PCR. We found that the polysome-bound mRNA levels of these markers were significantly decreased in METTL1-KD hiPSCs (Fig. 3d), indicating that the translation efficiency for pluripotency genes, including OCT4, Nanog, and SOX2, was remarkably decreased when METTL1 was silenced.

We next performed an anti-m^7^G northwestern assay to assess whether m^7^G modifications were altered in METTL1-KD hiPSCs. We observed that the m^7^G signal was lost in the RNAs of METTL1-KD cells (Fig. 3e, f). We also assessed whether the exogenous expression of METTL1 in METTL1-KD cells could rescue the defects in RNA methylation (Fig. 3g). We found that transfer of METTL1-WT, but not a catalytic inactive mutant, into METTL1-KD hiPSCs rescued m^7^G modifications on RNAs (Fig. 3h, i). Moreover, METTL1-WT also rescued the expression of OCT4, Nanog, and SOX2 in METTL1-KD cells (Fig. 3j). These findings indicated that METTL1 deficiency impacted hiPSC pluripotency by impairing the translation of three key pluripotency genes.

### METTL1 silencing accelerates hiPSC differentiation into embryoid bodies and promotes the expression of angiogenesis-related genes

**Fig. 4:**
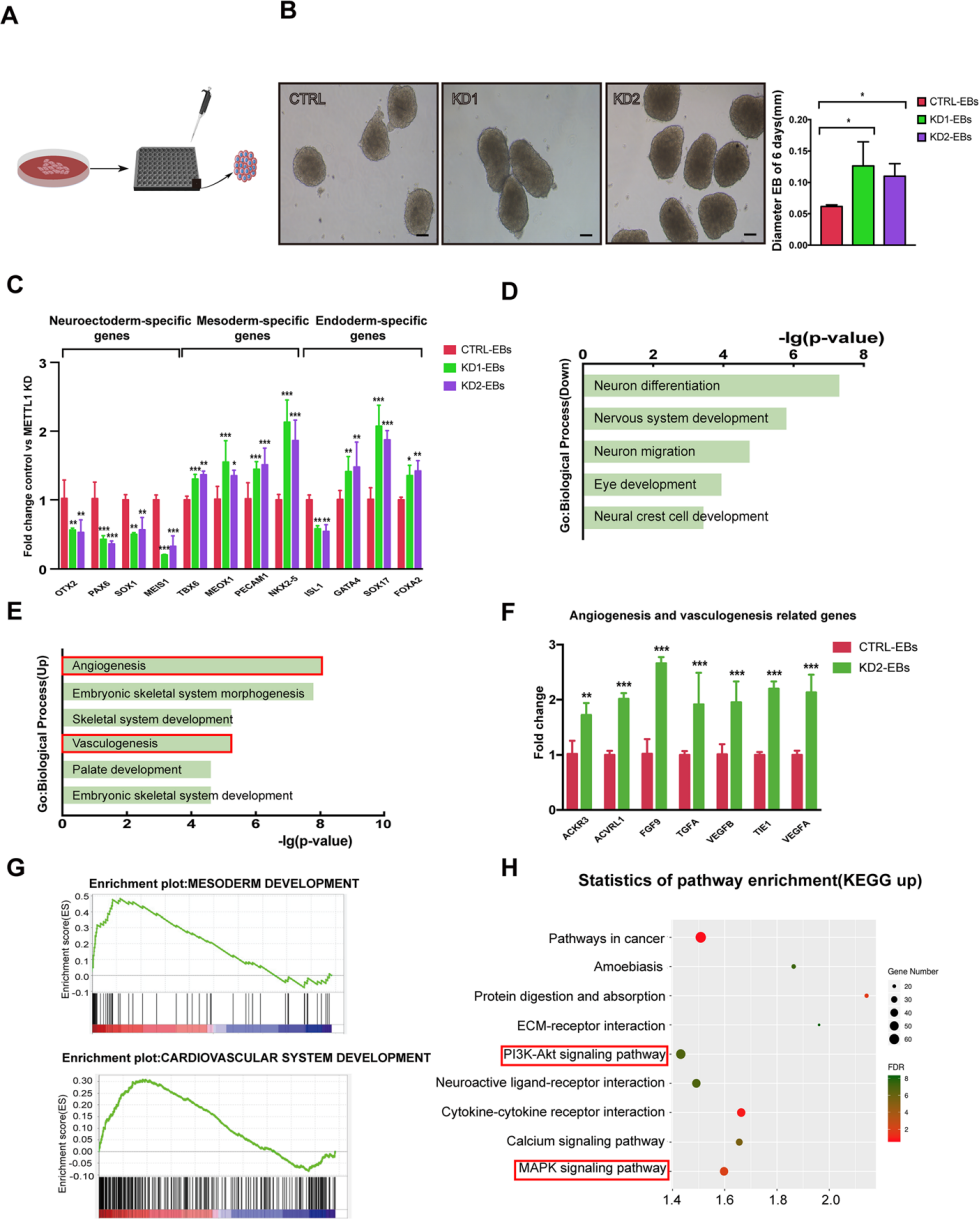
METTL1 silencing accelerates embryonic body formation and increases the expression of angiogenesis-related genes. **a** Schematic diagram showing the EB spheroid formation process. Cells were seeded on ultralow attachment 96-well spheroid microplates to induce EB formation. **b** Representative images and quantification of EB formation in control and METTL1-KD hiPSCs on day 6. Scale bar, 50 μm. **c** qRT-PCR analysis assessing the mRNA levels in control and METTL1-KD EBs. **d**, **e** Gene ontology analysis showing functional enrichment in biological process in genes downregulated and upregulated in METTL1-KD EBs. **f** qRT-PCR analysis assessing the mRNA levels of angiogenesis- and vasculogenesis-related genes in control and METTL1-KD EBs. **g** GSEA showing enrichment of the mesoderm and cardiovascular development gene signatures. *Y*-axis indicates enrichment score (ES), while the *X*-axis indicates positively and negatively correlated gene sets. **h** KEGG pathway analysis of genes upregulated in METTL1-KD EBs. Data are presented as mean ± SEM; *n* = 3; *P < 0.05, **P < 0.01; ***P < 0.001

HiPSC cells can spontaneously aggregate and form embryoid bodies (EBs) in vitro which comprised all three embryonic germ layers. Since our results suggested a potential role of METTL1 in hiPSC differentiation, we conducted an EB formation assay. To ensure the homogeneity of EBs, we used ultra-low attachment 96-well spheroid microplates to induce EB formation (Fig. 4a). HiPSCs formed EBs 6 days after seeding, and the EBs formed by METTL1-KD hiPSCs were significantly larger than those formed by control hiPSCs (Fig. 4b). We also assessed the expression of different lineage-specific markers and found that METTL1 knockdown resulted in increased expression of mesoderm- and endoderm-lineage markers, while the expression of ectoderm markers was reduced (Fig. 4c).

We also performed RNA-seq analysis in day 6 METTL1-KD-derived and control EBs. GO enrichment analysis of DEGs revealed enrichment of neuroectoderm markers, as well as genes involved in neuron differentiation and nervous system development among the downregulated in METTL1-KD EBs genes (Fig. 4d). Interestingly, a significant enrichment of genes involved in the regulation of angiogenesis and skeletal system development, which arises from mesoderm, was observed in the upregulated genes (Fig. 4e). Consistent with the RNA-seq findings, qRT-PCR confirmed the differential expression of mRNA expression of angiogenesis- and vasculogenesis-related genes (Fig. 4f). Moreover, we compared the change in gene expression levels for key pluripotent markers (Fig. S4a). We found that there were significant changes in multiple pluripotent factors regulating the stem cells, including SOX2/OCT4 and NANOG (Fig. S4b). According to literature reports, the upregulation of OCT4 and the downregulation of Nanog and SOX2 would force the stem cells to differentiate toward mesoderm [22–26], which coincide with our results. Gene set enrichment analysis (GSEA) indicated that mesoderm development and cardiovascular system development gene signatures were enriched in genes upregulated in METTL1-KD EBs (Fig. 4g). Furthermore, KEGG pathway analysis showed that numerous pathways associated with angiogenesis and vasculogenesis were significantly differentially regulated (Fig. 4h).

Overall, our data reveal that METTL1 silencing promotes embryonic differentiation in vitro and induces the expression of mesodermal markers, polarizing stem cells differentiation toward mesoderm lineages and promoting angiogenesis and vasculogenesis.

### METTL1 knockdown enhances teratoma formation in vivo by promoting cell proliferation and angiogenesis in nude mice

**Fig. 5:**
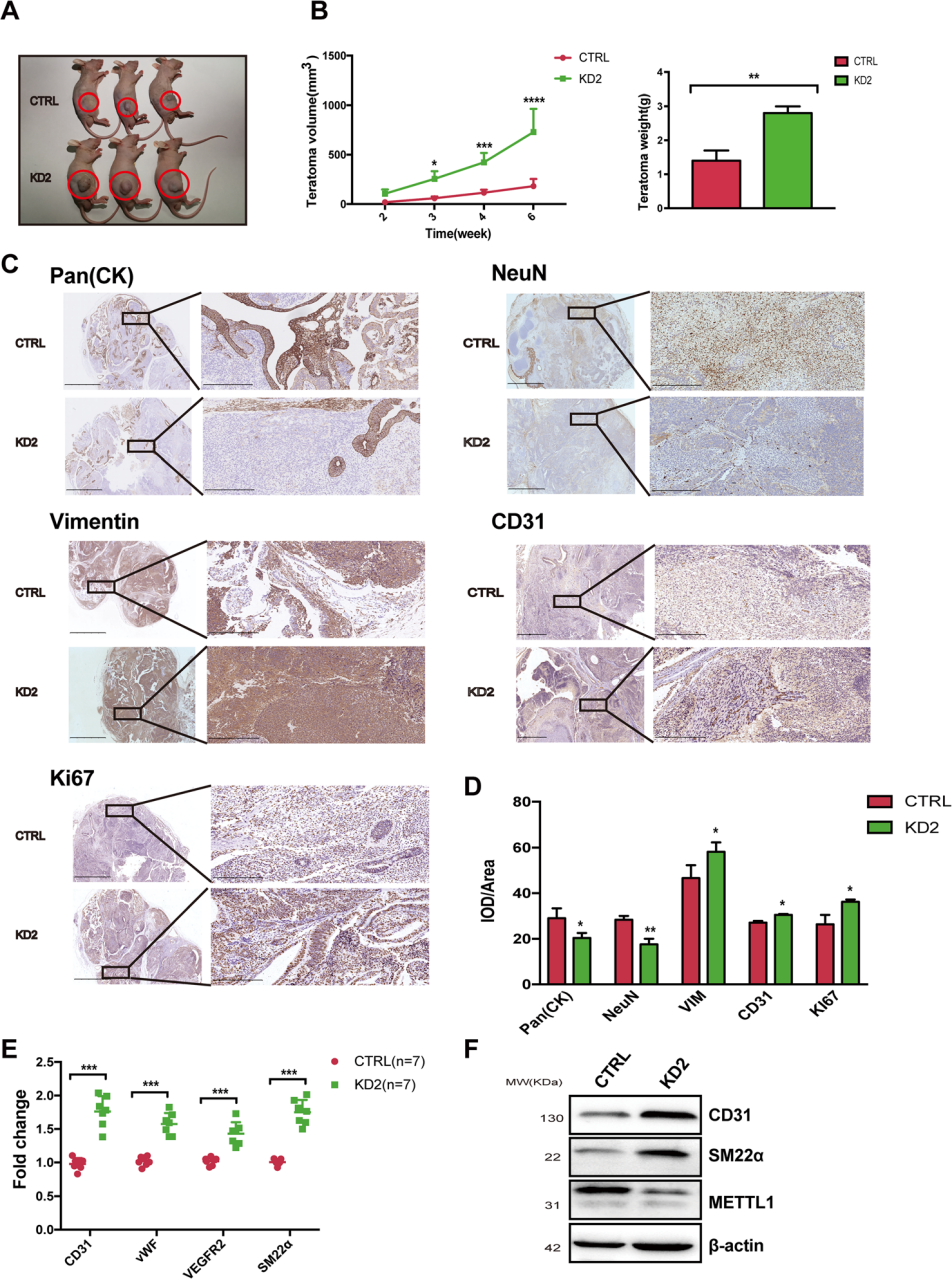
METTL1 knockdown accelerates teratoma development and angiogenesis in nude mice. **a** Teratomas formed 6 weeks post-injection in BALB/C nude mice. **b** Teratoma weight and volume were quantified; *n* = 5. **c**, **d** Immunohistochemical analysis of teratomas from METTL1-KD or control hiPSCs. Scale bar, 2.5 mm and 250 μm, respectively. Quantification of cell number is shown. **e** Quantification of CD31, vWF, VEGFR2, and SM22α mRNA levels in teratomas from METTL1-KD and control hiPSCs; *n* = 7 for tumor and control samples. **f** Representative western blot images for CD31, METTL1, and SM22α protein levels in teratomas from METTL1-KD hiPSCs and control hiPSCs; *n* = 3. Data are presented as mean ± SEM; *P < 0.05; ***P < 0.001; ****P < 0.0001

We then sought to assess whether teratoma formation in vivo was affected by METTL1 silencing. To this end, we injected control and METTL1-KD hiPSCs into the posterior limbs of BALB/C nude mice (Fig. S5a). Teratoma formation was observed at 6 weeks post-injection; we observed that METTL1-KD-derived teratomas had a significantly increased volume and weight compared to control teratomas (Fig. 5a, b; Fig. S5b).

We also investigated if the enhanced teratoma formation was due to increased differentiation of hiPSCs, as observed in the EB formation assay. H&E staining revealed that both the METTL1-KD-derived and control teratomas were fully differentiated into the three germ layers (Fig. S5c). These results indicated that hiPSCs maintained their ability to differentiate into cell types derived from all three primary germ layers despite METTL1 silencing. However, immunohistochemical analyses revealed a decreased number of cells expressing the ectodermal marker pan-cytokeratin METTL1-KD-derived teratomas, while the number of cells expressing the mesodermal marker vimentin was increased (Fig. 5c, d). Ki67 expression was also increased in METTL1-KD-derived teratomas, suggesting a higher number of proliferating cells. Moreover, a lower number of cells expressing the neural differentiation marker NeuN were detected in METTL1-KD-derived teratomas. In contrast, METTL1-KD-derived teratomas contained increased levels of the vascular endothelial cell marker CD31. qRT-PCR confirmed the increased levels of CD31, vWF, VEGFR2, and SM22α expression in METTL1-KD hiPSC-derived teratomas compared to control hiPSC-derived teratomas (Fig. 5e, f).

### METTL1 knockdown promotes angiogenesis in vivo

**Fig. 6:**
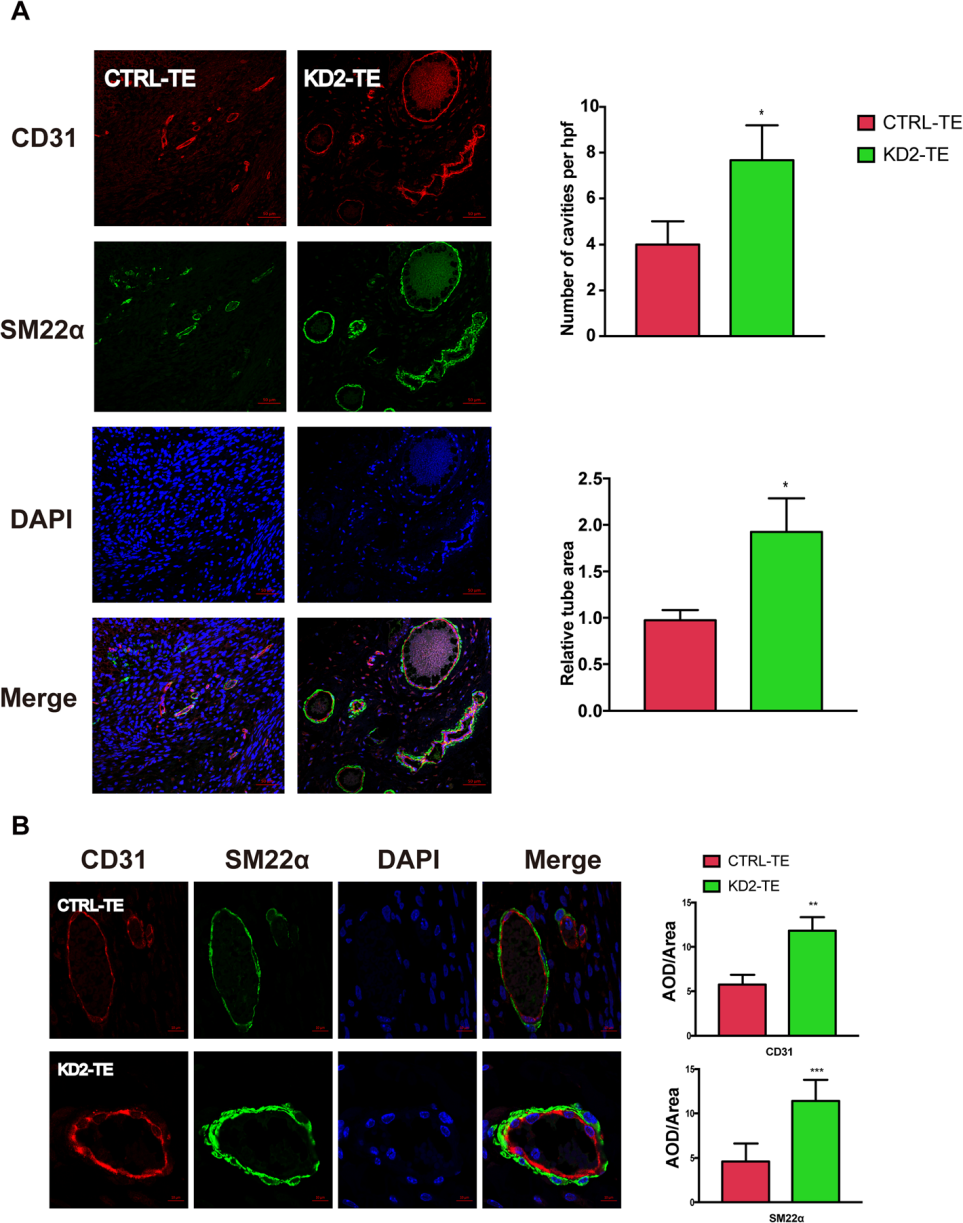
METTL1 silencing promotes angiogenesis and increased CD31 and SM22α expression in teratomas. **a** Representative images from control and METTL1-KD2 teratomas stained for CD31 (red), and SM22α (green); nuclei were counterstained with DAPI (blue). The number of cavities per hpf and relative tube area were determined using ImageJ software. Scale bar, 50 μm. **b** Representative images of vessels at high magnification (× 100) and quantification of CD31 and SM22α intensities in control and METTL1-KD teratomas; nuclei were counterstained with DAPI. Scale bar, 10 μm. Data are presented as mean ± SEM; *n* = 3; **P < 0.01, ***P < 0.001

To further confirm the increased neovascularization observed in METTL1-KD-derived teratomas, the expression of the surface markers of neovascular endothelial cells and vascular smooth muscle cells (VSMCs) CD31 and SM22α was quantified in sections from METTL1-KD-derived and control teratomas; the number of cavities per high power field (hpf) and the relative tube area were also calculated (Fig. 6a). Consistent with our previous findings, we found that METTL1 silencing enhanced the formatting of new blood vessels by endothelial cells and VSMCs. Furthermore, the numbers of CD31-positive ECs and SMA-positive VSMCs were significantly increased in METTL1-KD-derived EBs compared to the control EBs (Fig. 6b). These results indicated that METTL1 suppresses angiogenesis in vivo, as the formation of a vascular network was profoundly enhanced in METTL1-KD-derived EBs.

## Discussion

The use of iPSCs can overcome the ethical issues of ESCs and is considered as a promising alternative for drug screening, disease modeling, and development of clinical treatments [27–29]. The molecular mechanisms underlying self-renewal and maintenance of pluripotency in iPSCs have been extensively studied. The stem cell genome undergoes reversible epigenetic modifications that regulate gene expression and dictate cell development and differentiation [8, 11, 30, 31]. In our previous study, we reported that METTL1-deficient mouse ESCs display defective self-renewal and neural differentiation [13]. However, the exact molecular mechanisms by which METTL1 regulates pluripotency and differentiation of stem cells remained unknown.

RNA-seq analysis in METTL1-KD hiPSCs revealed an apparent downregulation of genes involved in maintaining stem cell self-renewal and pluripotency, including BMP4, FGF2, and WNT2. Simultaneously, various pathways associated with stem cell regulation were significantly downregulated [32, 33]. P53 signaling pathway has been considered to play an active role in promoting cell apoptosis and also plays a critical role in inhibiting the cell cycle of ESCs [34]. The upregulated p53 signaling pathway will lead to ESC differentiation [35, 36]. In our study, we found that although p53 signaling pathway is significantly inhibited in METTL1-KD hiPSCs, the self-renewal and stability of METTL1-KD hiPSCs were impaired. This may be due to the extensive influence of METTL1 on other signaling pathways that regulate hiPSCs. For example, the METTL1 regulated FGF/TGF-β/Wnt signalings play critical roles in maintaining the stability of stem cells [37, 38].

Changes in cell cycle regulation are also closely associated with stem cell fate [39, 40]. The cell cycle in stem cells is characterized by a faster G1 phase and extended S phase [41, 42], and hiPSCs maintain pluripotency through rapid self-renewal and inhibition of cell differentiation. Our findings suggested that METTL1 silencing resulted in cell cycle arrest in hiPSCs, with a potential impact on their self-renewal, which was in accordance with the previous studies that have demonstrated METTL1’s involvement in cell cycle regulation and pluripotency in mESCs [13].

Recent studies have shown that METTL1-mediated m^7^G methylation is not restricted to tRNA but also occurs on mRNAs and miRNAs, increasing translation efficiency and miRNA processing, respectively [43, 44]. Our polysome profiling revealed that suppression of METTL1-mediated m^7^G tRNA modifications in hiPSCs resulted in a marked decrease in translation efficiency of the stem cell transcription factors OCT4, SOX2, and Nanog. We showed that METTL1 knockdown reduced m^7^G tRNA modification and decreased the mRNA translation of stem cell transcription factors. However, whether m^7^G modification on RNAs other than tRNAs contributes to this process requires further investigation.

Next, we verify the role of METTL1 in differentiation in hiPSCs; we performed EB formation assay in vitro. The results showed that METTL1-KD hiPSCs tended to differentiate into mesoderm including neovascularization while inhibiting neuroectoderm differentiation. The latest research of our team also showed that METTL1 can promote the ability and function of hiPSCs to differentiate into endothelial progenitor cells (EPC) in vitro [45]. In addition, hiPSCs with pluripotency can differentiate into teratoma with three germ layers in mice [46, 47]. We found that teratomas derived from METTL1-KD hiPSCs exhibited lower levels of ectoderm markers and increased levels of mesoderm markers, consistent with our EB formation results and further supporting the role of METTL1 in the regulation of germ layer differentiation. Consistent with increased polarization toward mesoderm fate after METTL1 silencing, we observed increased vascular CD31^+^ blood vessels in METTL1-KD teratomas. The increased vascularization might explain the fact that the downregulation of METTL1 in hiPSCs enhanced in vivo tumor formation and progression. Angiogenesis is essential for embryonic development, as well as for tumor initiation and progression. Increased angiogenesis is often associated with enhanced cancer cell proliferation and tumor growth [48], which might explain why METTL1-KD teratomas exhibited increased cell proliferation and enhanced growth.

**Fig. 7:**
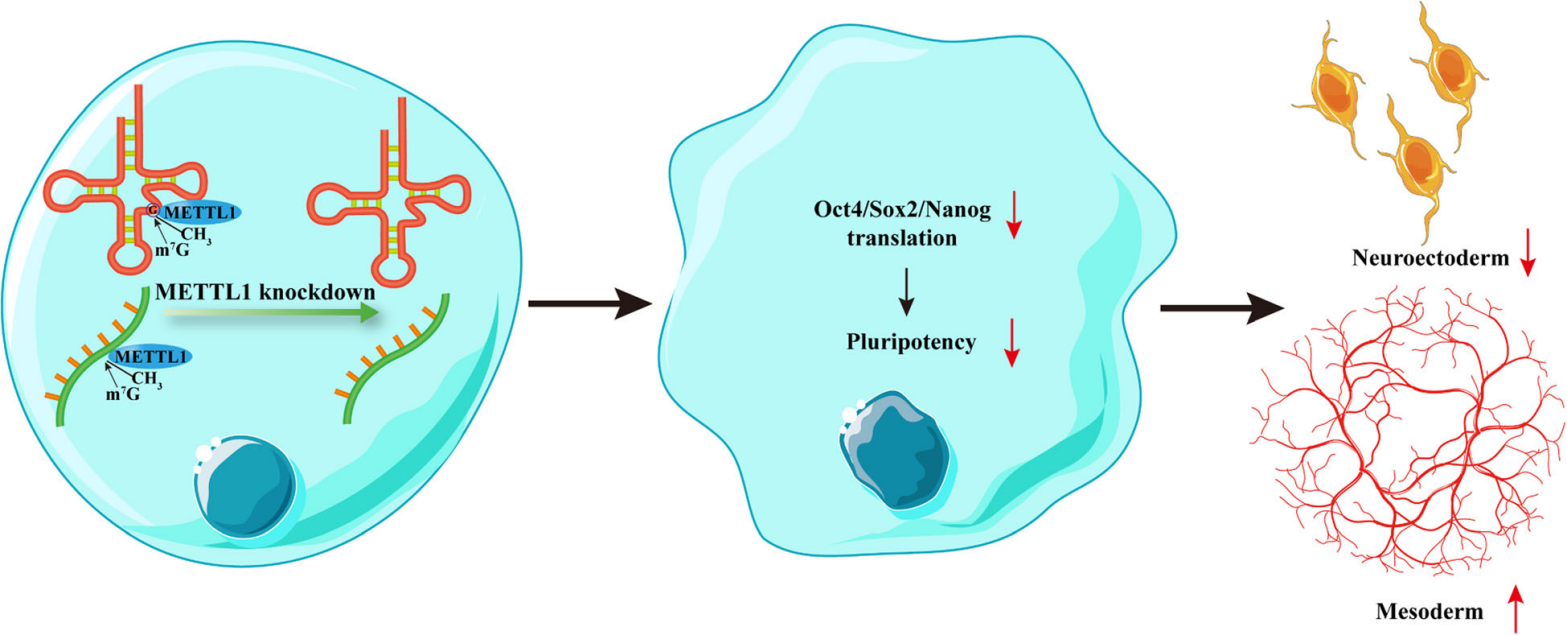
A graphic for the role of METTL1 in regulating stem cell pluripotency and differentiation. METTL1-mediated m^7^G methylation is essential for the translation of pluripotency transcription factors. METTL1 knockdown impairs neurectoderm formation while accelerates mesoderm differentiation and vasculogenesis/angiogenesis

Taken together, the results of our study highlighted the role of METTL1 in the processes of pluripotency maintenance, self-renewal, and differentiation of hiPSCs. Our findings indicated, for the first time, the physiological function of METTL1 in hiPSCs and that METTL1-mediated m^7^G methylation is essential for translation of pluripotency transcription factors. Our findings also suggested that METTL1 suppresses angiogenesis by inhibiting mesoderm differentiation (Fig. 7). More importantly, since hiPSCs offer a powerful tool to model human ontogenetic processes in vivo and in vitro [46, 49], the findings of this study have strong implications in human embryo development research. However, the molecular mechanisms underlying the regulation of METTL1 during these processes require further investigation. Understanding how m^7^G on mRNAs and tRNAs are dynamically regulated in stem cell differentiation may provide improved strategies for the treatment of neurological and vascular diseases.

## Conclusion

This study aimed at assessing the biological relevance and exact mechanisms by which METTL1 regulates pluripotency and differentiation in human induced pluripotent stem cells (hiPSCs). In summary, the results of this study make a significant contribution to the literature for our findings demonstrated that METTL1 is essential in maintaining pluripotency in human stem cells. We found METTL1 silencing resulted in alterations in the global m^7^G profile in hiPSCs, as well as reduced the translational efficiency of stem cell marker genes. METTL1-KD hiPSCs exhibited reduced pluripotency with slower cell cycling. METTL1 silencing also skewed hiPSC differentiation toward the mesoderm fate and suppressed neuroectoderm differentiation both in vivo and in vitro.

## Supplementary information

**Additional file 1: Figure S1.** Identification of METTL1-modulated genes by RNA-seq. a qRT-PCR and WB analyses of METTL1 expression levels in KD, WT, and control cells. b Heatmap showing differentially expressed genes involved in neurectoderm development. Each lane corresponds to an independent biological sample. Upregulated and downregulated genes are shown in red and blue, respectively. **c** KEGG pathway analysis of genes upregulated in METTL1-KD hiPSCs.

**Additional file 2: Figure S2.** METTL1 regulates cell cycle progression in hiPSCs and morphology of ESCs. a qRT-PCR analysis assessing the mRNA levels of cell cycle markers in METTL1-KD and control hiPSCs. b Western blot assessing the protein levels of cell cycle regulators. Images are representative of three independent experiments. c Colony-formation assay using METTL1-KD and control hESCs. Representative images and colony quantification data are shown. Data are presented as mean ± SEM; *n* = 3; *, *P* < 0.05; **, *P* < 0.01; ***, *P* < 0.001.

**Additional file 3: Figure S3.** METTL1-mediated m^7^G methylation is required for OCT4, Nanog, and SOX2 expression in ESCs. (a- b) qRT-PCR and WB assessing the mRNA and protein levels of Oct4, Nanog, and Sox2 in METTL1-KD and control H1ESCs (a) and H9ESCs (b) cells. Data are presented as mean ± SEM; n = 3; ***, *P* < 0.001.

**Additional file 4: Figure S4.** Expression of pluripotency genes on METTL1-KD and control hiPSCs derived EBs. (a) Heatmap showing DEGs involved in stem cells pluripotency pathways. Each lane corresponds to an independent biological sample. Scale bar: log2 FPKM. (b) qRT-PCR analysis assessing the mRNA levels of OCT4, Nanog, and SOX2 in control and METTL1-KD derived EBs. Data are presented as mean ± SEM; n = 3; **, *P* < 0.01; ***, *P* < 0.001.

**Additional file 5: Figure S5.** METTL1 knockdown promotes teratoma differentiation in nude mice. a Schematic diagram summarizing the procedure of subcutaneous injection of matrigel solution containing METTL1-KD hiPSCs or control hiPSCs into 5-week-old BALB/C nude mice. b Tumors were isolated 6 weeks after injection. c Haematoxylin and eosin (HE) staining of the teratoma tissues. The development of three germ layers (ectoderm, mesoderm, and endoderm) was evident in the teratoma tissue.

**Additional file 6: Table S1.** List of culture mediums and chemical reagents used in this study. **Table S2.** List of primary and secondary antibodies used. **Table S3.** Cell lines used in this study. **Table S4.** Sequences of the primes used in this study. **Table S5.** Components of polysome cell extraction buffer.

## Data Availability

The datasets used and/or analyzed during the current study are available from the corresponding author on reasonable request.

## Abbreviations

FBS: Fetal bovine serum
KSR: Knockout-serum replacement
AP: Alkaline phosphatase
DEGs: Differentially expressed genes
EBs: Embryoid bodies
ESCs: Embryonic stem cells
FDR: False discovery rate
RPKM: Reads per kilobase of transcript per million mapped reads
GSEA: Gene set enrichment analysis
GO: Gene ontology
HE: Hematoxylin and eosin staining
IHC: Immunohistochemistry
KEGG: Kyoto Encyclopedia of Genes and Genomes
m^7^G: 7-Methylguanosine
2-Me: 2-Mercaptoethanol
NEAA: Non-essential amino acid
MeRIP-Seq: Methylated RNA immunoprecipitation sequence
RT-PCR: Quantitative real-time PCR
RNA-seq: RNA sequence

## References

[1] Takahashi K, Tanabe K, Ohnuki M, Narita M, Ichisaka T, Tomoda K, Yamanaka S (2007). Induction of pluripotent stem cells from adult human fibroblasts by defined factors. Cell.

[2] Yu J, Vodyanik MA, Smuga-Otto K, Antosiewicz-Bourget J, Frane JL, Tian S, Nie J, Jonsdottir GA, Ruotti V, Stewart R (2007). Induced pluripotent stem cell lines derived from human somatic cells. Science.

[3] Wu SM, Hochedlinger K (2011). Harnessing the potential of induced pluripotent stem cells for regenerative medicine. Nat Cell Biol.

[4] Stadtfeld M, Hochedlinger K (2010). Induced pluripotency: history, mechanisms, and applications. Genes Dev.

[5] Thomson M, Liu SJ, Zou LN, Smith Z, Meissner A, Ramanathan S (2011). Pluripotency factors in embryonic stem cells regulate differentiation into germ layers. Cell.

[6] Kuppusamy KT, Sperber H, Ruohola-Baker H (2013). MicroRNA regulation and role in stem cell maintenance, cardiac differentiation and hypertrophy. Curr Mol Med.

[7] Warren L, Lin C (2019). mRNA-based genetic reprogramming. Mol Ther.

[8] Jambhekar A, Dhall A, Shi Y (2019). Roles and regulation of histone methylation in animal development. Nat Rev Mol Cell Biol.

[9] Srinageshwar B, Maiti P, Dunbar GL, et al. Role of epigenetics in stem cell proliferation and differentiation: implications for treating neurodegenerative diseases. Int J Mol Sci. 2016;17(2):199-214. 10.3390/ijms17020199 PMC4783933 26848657

[10] Ren R, Ocampo A, Liu GH, Izpisua Belmonte JC (2017). Regulation of stem cell aging by metabolism and epigenetics. Cell Metab.

[11] Wu Y, Zhou C, Yuan Q (2018). Role of DNA and RNA N6-adenine methylation in regulating stem cell fate. Curr Stem Cell Res Ther.

[12] Shaheen R, Abdel-Salam GM, Guy MP, Alomar R, Abdel-Hamid MS, Afifi HH, Ismail SI, Emam BA, Phizicky EM, Alkuraya FS (2015). Mutation in WDR4 impairs tRNA m(7) G46 methylation and causes a distinct form of microcephalic primordial dwarfism. Genome Biol.

[13] Lin S, Liu Q, Lelyveld VS, Choe J, Szostak JW, Gregory RI (2018). Mettl1/Wdr4-mediated m(7) G tRNA methylome is required for normal mRNA translation and embryonic stem cell self-renewal and differentiation. Mol Cell.

[14] Ingolia NT, Lareau LF, Weissman JS (2011). Ribosome profiling of mouse embryonic stem cells reveals the complexity and dynamics of mammalian proteomes. Cell.

[15] Sampath P, Pritchard DK, Pabon L, Reinecke H, Schwartz SM, Morris DR, Murry CE (2008). A hierarchical network controls protein translation during murine embryonic stem cell self-renewal and differentiation. Cell Stem Cell.

[16] Subramanian A, Guo B, Marsden MD, Galic Z, Kitchen S, Kacena A, Brown HJ, Cheng G, Zack JA (2009). Macrophage differentiation from embryoid bodies derived from human embryonic stem cells. J Stem Cells.

[17] Itskovitz-Eldor J, Schuldiner M, Karsenti D, Eden A, Yanuka O, Amit M, Soreq H, Benvenisty N (2000). Differentiation of human embryonic stem cells into embryoid bodies compromising the three embryonic germ layers. Mol Med.

[18] da Huang W, Sherman BT, Lempicki RA (2009). Bioinformatics enrichment tools: paths toward the comprehensive functional analysis of large gene lists. Nucleic Acids Res.

[19] Qu Q, Li D, Louis KR, Li X, Yang H, Sun Q, Crandall SR, Tsang S, Zhou J, Cox CL (2014). High-efficiency motor neuron differentiation from human pluripotent stem cells and the function of Islet-1. Nat Commun.

[20] Laugwitz KL, Moretti A, Lam J, Gruber P, Chen Y, Woodard S, Lin LZ, Cai CL, Lu MM, Reth M (2005). Postnatal isl1+ cardioblasts enter fully differentiated cardiomyocyte lineages. Nature.

[21] Lutolf MP, Gilbert PM, Blau HM (2009). Designing materials to direct stem-cell fate. Nature.

[22] Ding J, Xu H, Faiola F, Ma'ayan A, Wang J (2012). Oct4 links multiple epigenetic pathways to the pluripotency network. Cell Res.

[23] Lengner CJ, Welstead GG, Jaenisch R (2008). The pluripotency regulator Oct4: a role in somatic stem cells?. Cell Cycle.

[24] Loh YH, Wu Q, Chew JL, Vega VB, Zhang W, Chen X, Bourque G, George J, Leong B, Liu J (2006). The Oct4 and Nanog transcription network regulates pluripotency in mouse embryonic stem cells. Nat Genet.

[25] Masui S, Nakatake Y, Toyooka Y, Shimosato D, Yagi R, Takahashi K, Okochi H, Okuda A, Matoba R, Sharov AA (2007). Pluripotency governed by Sox2 via regulation of Oct3/4 expression in mouse embryonic stem cells. Nat Cell Biol.

[26] Simandi Z, Horvath A, Wright LC, Cuaranta-Monroy I, De Luca I, Karolyi K, Sauer S, Deleuze JF, Gudas LJ, Cowley SM (2016). OCT4 acts as an integrator of pluripotency and signal-induced differentiation. Mol Cell.

[27] Salaris F, Rosa A (2019). Construction of 3D in vitro models by bioprinting human pluripotent stem cells: challenges and opportunities. Brain Res.

[28] Lee CT, Bendriem RM, Wu WW, Shen RF (2017). 3D brain organoids derived from pluripotent stem cells: promising experimental models for brain development and neurodegenerative disorders. J Biomed Sci.

[29] Flaherty EK, Brennand KJ (2017). Using hiPSCs to model neuropsychiatric copy number variations (CNVs) has potential to reveal underlying disease mechanisms. Brain Res.

[30] Ankam S, Rovini A, Baheti S, Hrstka R, Wu Y, Schmidt K, Wang H, Madigan N, Koenig LS, Stelzig K (2019). DNA methylation patterns in human iPSC-derived sensory neuronal differentiation. Epigenetics.

[31] Dovey OM, Foster CT, Cowley SM (2010). Histone deacetylase 1 (HDAC1), but not HDAC2, controls embryonic stem cell differentiation. Proc Natl Acad Sci U S A.

[32] Wu M, Chen G, Li YP (2016). TGF-beta and BMP signaling in osteoblast, skeletal development, and bone formation, homeostasis and disease. Bone Res.

[33] Mohammadnia A, Yaqubi M, Pourasgari F, Neely E, Fallahi H, Massumi M (2016). Signaling and gene regulatory networks governing definitive endoderm derivation from pluripotent stem cells. J Cell Physiol.

[34] Hong H, Takahashi K, Ichisaka T, Aoi T, Kanagawa O, Nakagawa M, Okita K, Yamanaka S (2009). Suppression of induced pluripotent stem cell generation by the p53-p21 pathway. Nature.

[35] Lee DF, Su J, Ang YS, Carvajal-Vergara X, Mulero-Navarro S, Pereira CF, Gingold J, Wang HL, Zhao R, Sevilla A (2012). Regulation of embryonic and induced pluripotency by aurora kinase-p53 signaling. Cell Stem Cell.

[36] Han MK, Song EK, Guo Y, Ou X, Mantel C, Broxmeyer HE (2008). SIRT1 regulates apoptosis and Nanog expression in mouse embryonic stem cells by controlling p53 subcellular localization. Cell Stem Cell.

[37] Van Camp JK, Beckers S, Zegers D, Van Hul W (2014). Wnt signaling and the control of human stem cell fate. Stem Cell Rev Rep.

[38] Coutu DL, Galipeau J (2011). Roles of FGF signaling in stem cell self-renewal, senescence and aging. Aging (Albany NY).

[39] Calder A, Roth-Albin I, Bhatia S, Pilquil C, Lee JH, Bhatia M, Levadoux-Martin M, McNicol J, Russell J, Collins T (2013). Lengthened G1 phase indicates differentiation status in human embryonic stem cells. Stem Cells Dev.

[40] Sela Y, Molotski N, Golan S, Itskovitz-Eldor J, Soen Y (2012). Human embryonic stem cells exhibit increased propensity to differentiate during the G1 phase prior to phosphorylation of retinoblastoma protein. Stem Cells.

[41] Pauklin S, Vallier L (2013). The cell-cycle state of stem cells determines cell fate propensity. Cell.

[42] Dalton S (2015). Linking the cell cycle to cell fate decisions. Trends Cell Biol.

[43] Zhou M, Wang H, Zeng X, Yin P, Zhu J, Chen W, Li X, Wang L, Wang L, Liu Y (2019). Mortality, morbidity, and risk factors in China and its provinces, 1990-2017: a systematic analysis for the Global Burden of Disease Study 2017. Lancet.

[44] Pandolfini L, Barbieri I, Bannister AJ, Hendrick A, Andrews B, Webster N, Murat P, Mach P, Brandi R, Robson SC (2019). METTL1 promotes let-7 MicroRNA processing via m7G methylation. Mol Cell.

[45] Deng Y, Zhou Z, Lin S, Yu B. METTL1 limits differentiation and functioning of EPCs derived from human-induced pluripotent stem cells through a MAPK/ERK pathway. Biochem Biophys Res Commun. 2020;527(3):791-8. 10.1016/j.bbrc.2020.04.115 32430183

[46] Nelakanti RV, Kooreman NG, Wu JC: Teratoma formation: a tool for monitoring pluripotency in stem cell research. Curr Protoc Stem Cell Biol 2015, 32:4A 8 1–17. 10.1002/9780470151808.sc04a08s32 PMC4402211 25640819

[47] Kooreman NG, Wu JC (2010). Tumorigenicity of pluripotent stem cells: biological insights from molecular imaging. J R Soc Interface.

[48] Carmeliet P, Jain RK (2011). Molecular mechanisms and clinical applications of angiogenesis. Nature.

[49] Bulic-Jakus F, Katusic Bojanac A, Juric-Lekic G, Vlahovic M, Sincic N (2016). Teratoma: from spontaneous tumors to the pluripotency/malignancy assay. Wiley Interdiscip Rev Dev Biol.

