# Supplementary figures and images for: METTL1-mediated m7G methylation maintains pluripotency in human stem cells and limits mesoderm differentiation and vascular development
Source: Stem Cell Res Ther. 2020 Jul 22;11:306. doi: 10.1186/s13287-020-01814-4 (PMC7374972; doi:10.1186/s13287-020-01814-4)

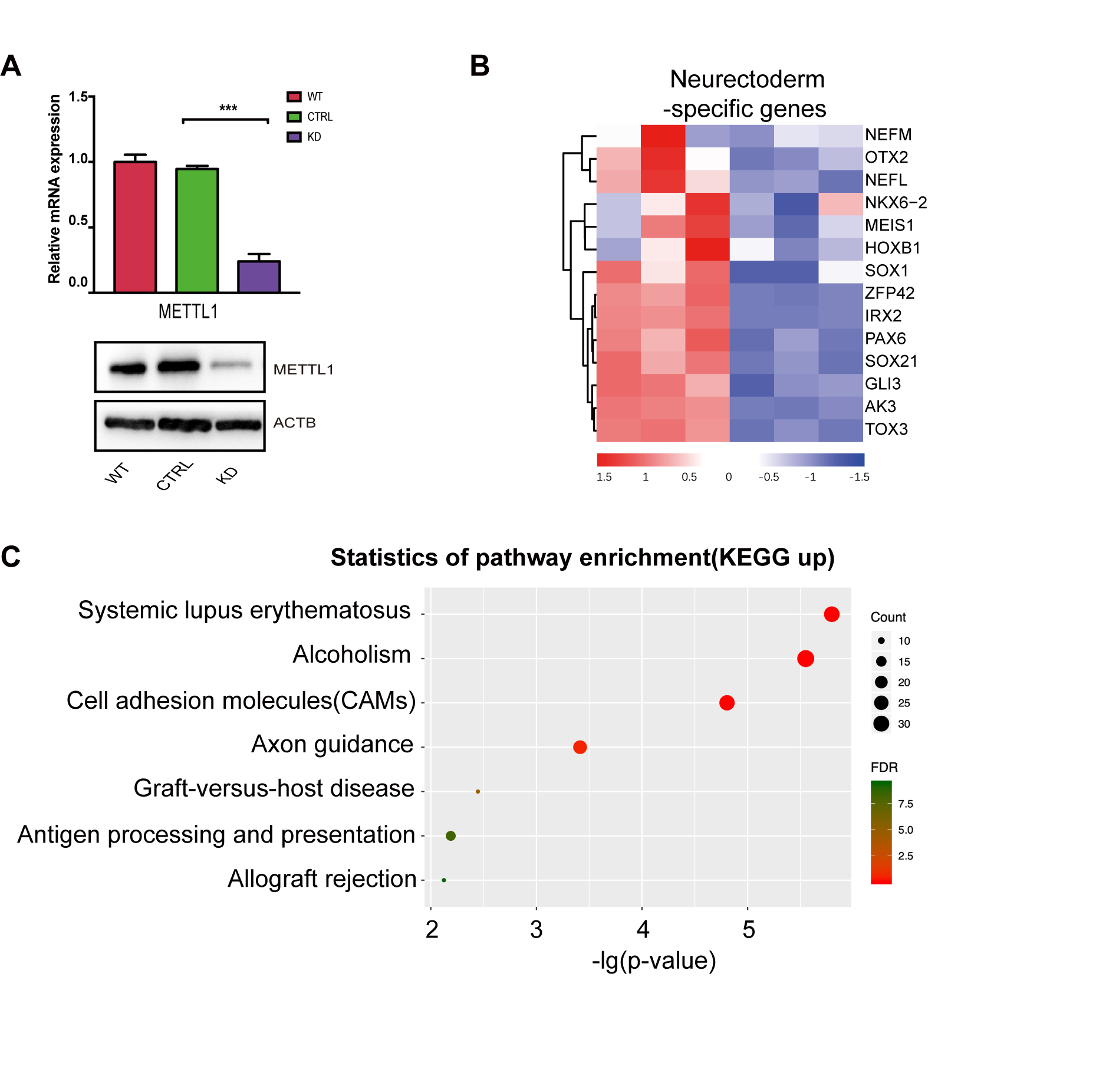

Supplement: Supplementary file 1 — Additional file 1: Figure S1. Identification of METTL1-modulated genes by RNA-seq. a qRT-PCR and WB analyses of METTL1 expression levels in KD, WT, and control cells. b Heatmap showing differentially expressed genes involved in neurectoderm development. Each lane corresponds to an independent biological sample. Upregulated and downregulated genes are shown in red and blue, respectively. c KEGG pathway analysis of genes upregulated in METTL1-KD hiPSCs. [file 13287_2020_1814_MOESM1_ESM.tif]

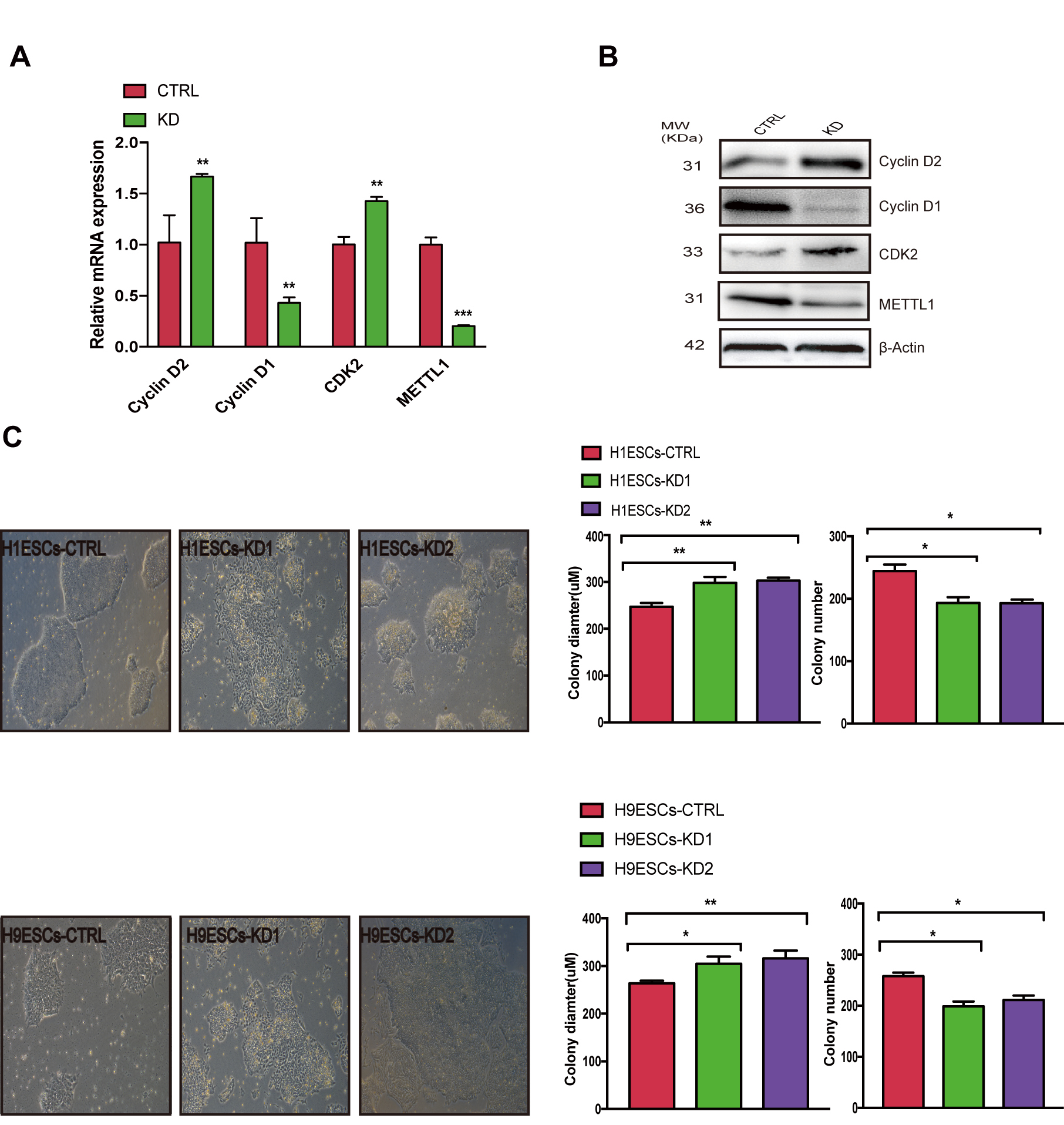

Supplement: Supplementary file 2 — Additional file 2: Figure S2. METTL1 regulates cell cycle progression in hiPSCs and morphology of ESCs. a qRT-PCR analysis assessing the mRNA levels of cell cycle markers in METTL1-KD and control hiPSCs. b Western blot assessing the protein levels of cell cycle regulators. Images are representative of three independent experiments. c Colony-formation assay using METTL1-KD and control hESCs. Representative images and colony quantification data are shown. Data are presented as mean ± SEM; n = 3; *, P < 0.05; **, P < 0.01; ***, P < 0.001. [file 13287_2020_1814_MOESM2_ESM.tif]

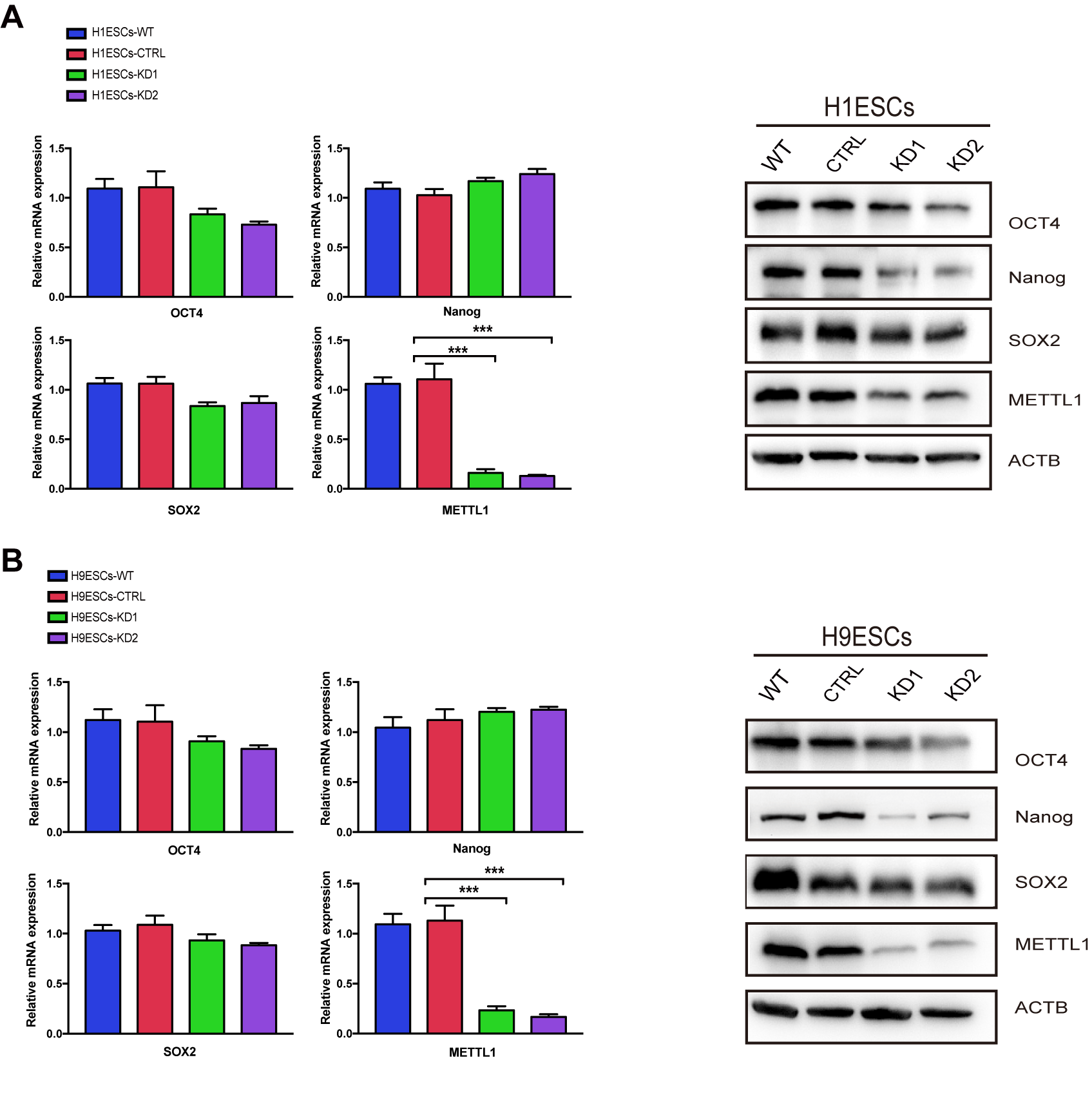

Supplement: Supplementary file 3 — Additional file 3: Figure S3. METTL1-mediated m7G methylation is required for OCT4, Nanog, and SOX2 expression in ESCs. (a- b) qRT-PCR and WB assessing the mRNA and protein levels of Oct4, Nanog, and Sox2 in METTL1-KD and control H1ESCs (a) and H9ESCs (b) cells. Data are presented as mean ± SEM; n = 3; ***, P < 0.001. [file 13287_2020_1814_MOESM3_ESM.tif]

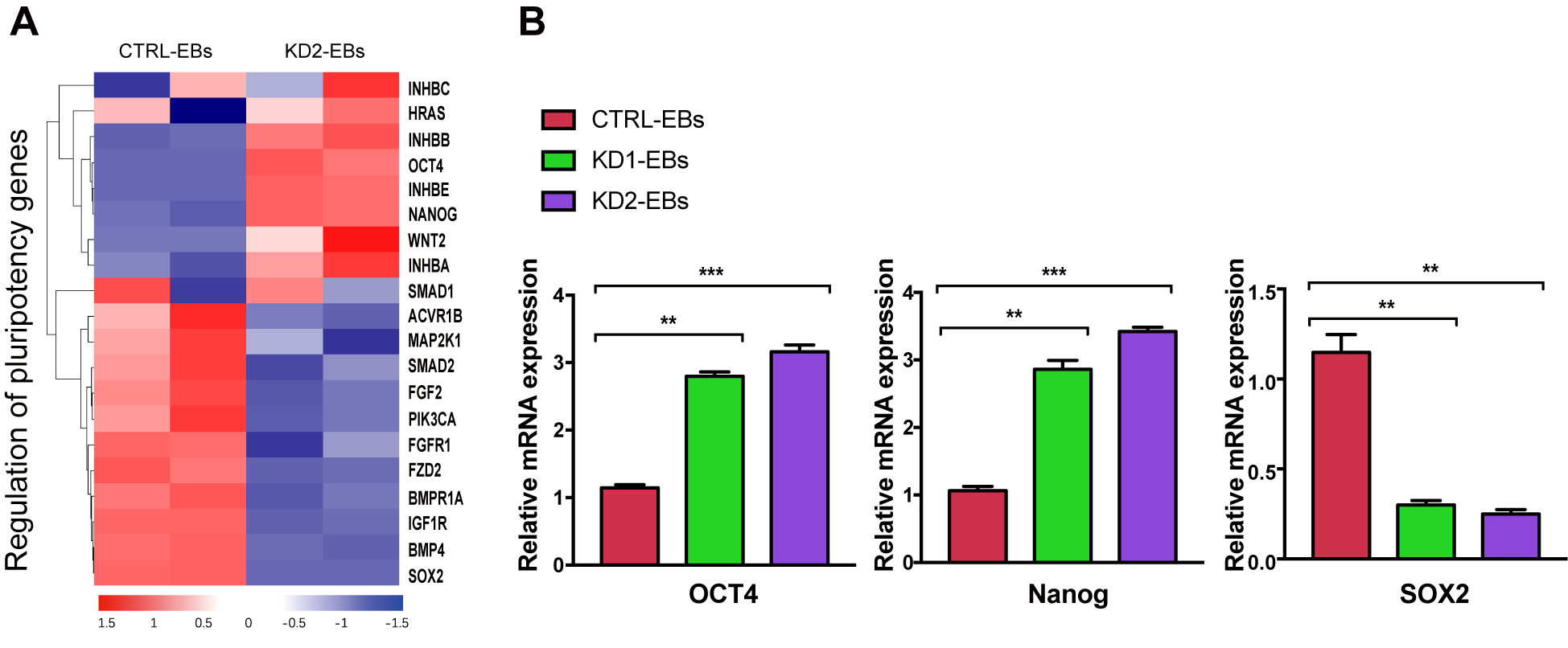

Supplement: Supplementary file 4 — Additional file 4: Figure S4. Expression of pluripotency genes on METTL1-KD and control hiPSCs derived EBs. (a) Heatmap showing DEGs involved in stem cells pluripotency pathways. Each lane corresponds to an independent biological sample. Scale bar: log2 FPKM. (b) qRT-PCR analysis assessing the mRNA levels of OCT4, Nanog, and SOX2 in control and METTL1-KD derived EBs. Data are presented as mean ± SEM; n = 3; **, P < 0.01; ***, P < 0.001. [file 13287_2020_1814_MOESM4_ESM.tif]

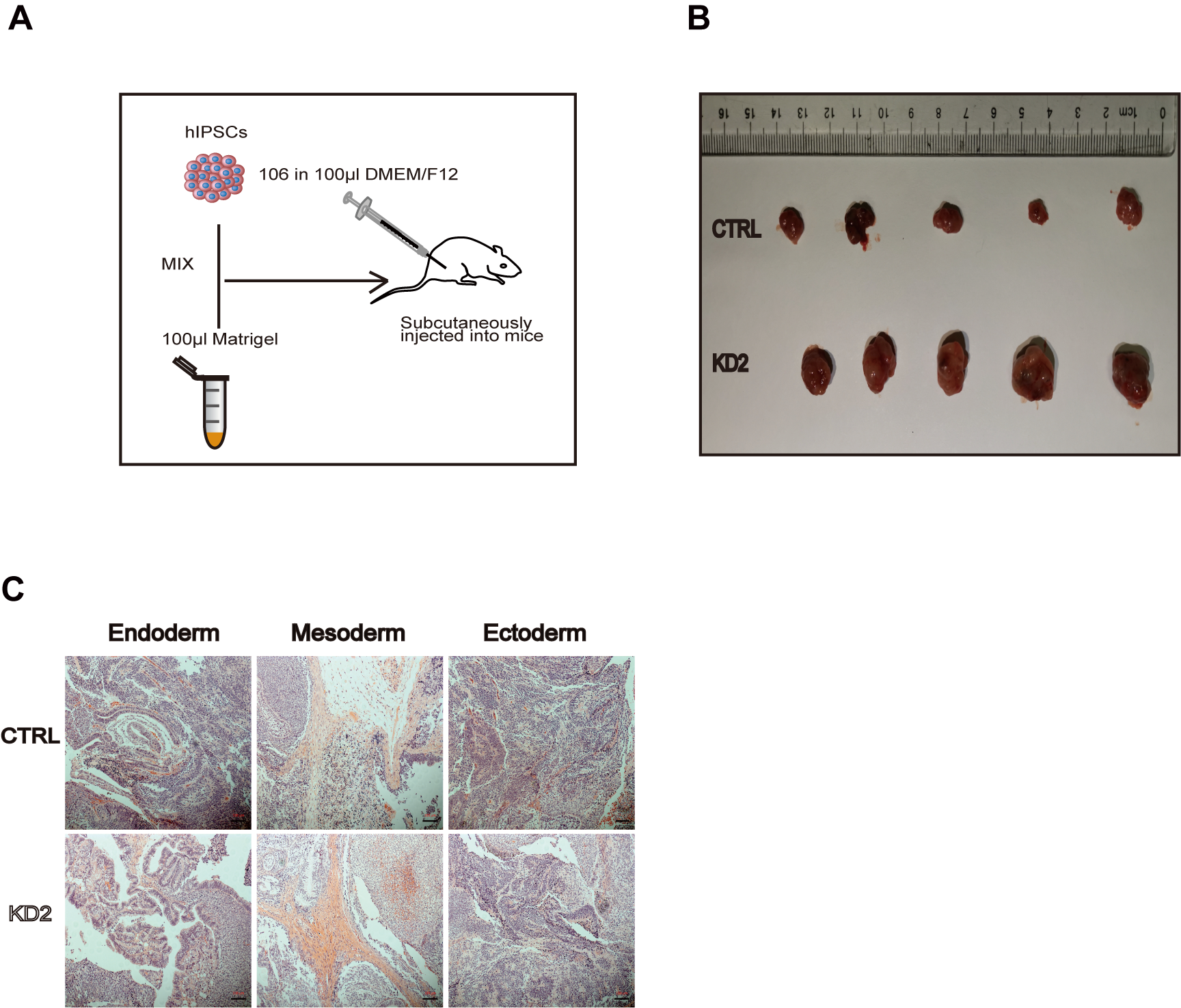

Supplement: Supplementary file 5 — Additional file 5: Figure S5. METTL1 knockdown promotes teratoma differentiation in nude mice. a Schematic diagram summarizing the procedure of subcutaneous injection of matrigel solution containing METTL1-KD hiPSCs or control hiPSCs into 5-week-old BALB/C nude mice. b Tumors were isolated 6 weeks after injection. c Haematoxylin and eosin (HE) staining of the teratoma tissues. The development of three germ layers (ectoderm, mesoderm, and endoderm) was evident in the teratoma tissue. [file 13287_2020_1814_MOESM5_ESM.tif]
