# Supplementary material for: METTL1-mediated m7G methylation maintains pluripotency in human stem cells and limits mesoderm differentiation and vascular development
Source: Stem Cell Res Ther. 2020 Jul 22;11:306. doi: 10.1186/s13287-020-01814-4 (PMC7374972; doi:10.1186/s13287-020-01814-4)
Supplement: Supplementary file 6 — Additional file 6: Table S1. List of culture mediums and chemical reagents used in this study. Table S2. List of primary and secondary antibodies used. Table S3. Cell lines used in this study. Table S4. Sequences of the primes used in this study. Table S5. Components of polysome cell extraction buffer. [file 13287_2020_1814_MOESM6_ESM.docx]

**Title: METTL1-mediated m^7^G methylation maintains pluripotency in human stem cells and limits mesoderm differentiation and vascular development**

Yujie Deng^1,2^, Zhongyang Zhou^1^, Weidong Ji^1^, Shuibin Lin^1^*, Min Wang^1^*

^1^Center for Translational Medicine, The First Affiliated Hospital, Sun Yat-sen University, Guangzhou, 510080, China

^2^Department of Rehabilitation Medicine, The Sixth Affiliated Hospital, Sun Yat-sen University, Guangzhou, 510000, China

*Correspondence: [mikewang388@gmail.com](mailto:mikewang388@gmail.com); [linshb6@mail.sysu.edu.cn](mailto:linshb6@mail.sysu.edu.cn)

**Running title**: METTL1 regulates the fate of human stem cells

**Table S1.** List of culture mediums and chemical reagents used in this study

| Name | Vendor/Source | Catalog | Concentration |
| --- | --- | --- | --- |
| mTeSR1 | Stem cell Technologies | 85850 | / |
| DMEM | Invitrogen | C11995500CP | / |
| KO-DMEM | Gibco | 10829018 | / |
| FBS | Merck | F8687 | 10% |
| KSR | Gibco | 10828028 | 10 % |
| Matrigel | BD Biosciences | 354230 | 50 μL/cm^2^ |
| Polybrene | Sigma | TR-1003-G | 8 μg/ml |
| Puromycin | Solarbio | P8230 | 1 μg /ml |
| TRIzol | Thermo Fisher Scientific | 15596026 | / |
| SYBR Green PCR Master Mix | Applied Biosystems | A25742 | / |
| Accutase | Stemcell Technologies | # 07920 | / |
| Propidium Iodide | eBioscience | P3566 | / |
| EDTA | Gibco | AM9260G | 0.5 mM |
| NEAA | Gibco | 11140050 | 1% |
| L-Glutamine | Sigma-Aldrich | G7513 | 1 mM |
| 2-Me | Sigma-Aldrich | SML0640 | 50 µM |
| Y-27632 | Sigma-Aldrich | #Y0503 | 10 µM |
| MOPS | Sigma-Aldrich | M1254 | 50 mM |
| Cycloheximide | Sigma-Aldrich | 5087390001 | 100 μg/mL |
| RNaseOUT | Thermo Fisher Scientific | 10777019 | 200 U |
| PMSF | Thermo Fisher Scientific | 36978 | 2 mM |
| heparin | Sigma-Aldrich | H3149 | 1 mg/ml |
| Benzamine | Sigma-Aldrich | 422827 | 1 μM |

**Table S2.** List of primary and secondary antibodies used

| Target antigen | Vendor/Source | Catalog | Concentration |
| --- | --- | --- | --- |
| METTL1 | Proteintech | #14994-1-AP | 0.3 μg/mL |
| OCT4 | Millpore | #MABD76 | 0.5 μg/mL |
| SOX2 | CST | #23064 | 0.2 μg/mL |
| NANOG | Abcam | #ab109250 | 0.369 ng/mL |
| FLAG | Millpore | #MAB3118 | 0.5 μg/mL |
| ACTB | CST | #4970 | 80 ng/mL |
| α-Tubulin | Santa Cruz | #sc-8035 | 0.2 μg/mL |
| Mouse IgG | CST | #7076 | 0.2 μg/mL |
| Rabbit IgG | CST | #7074 | 0.2 μg/mL |
| Alexa Fluor 488 | Invitrogen | A-21202 | 1 μg/mL |
| Alexa Fluor 594 | Invitrogen | A-21207 | 1 μg/mL |
| m^7^G | MBL | #RN017M | 0.001 mg/mL |
| NeuN | Sigma | ZRB377 | 2 μg/mL |
| Ki67 | CST | #9449S | 3 μg/mL |
| Pan (Cytokeratin) | ThermoFisher | # MS-343-P0 | 500 ng/mL |
| Vimentin | CST | #5741T | 2 μg/mL |
| CD31 | Abcam | #ab56299 | 2 μg/mL |
| SM22α | Abcam | #14106 | 3 μg/mL |

**Table S3.** Cell lines used in this study

| Name | Vendor or Source |
| --- | --- |
| hiPSC (DYR0100 cells ) | Stem Cell Bank, Chinese Academy of Science |
| H1ESCs | Stem Cell Bank, Chinese Academy of Science |
| H9ESCs | Stem Cell Bank, Chinese Academy of Science |
| 293T | ATCC |

**Table S4.** Sequences of the primes used in this study

| Gene | Primer Sequence |
| --- | --- |
| β-Actin | F: 5ˊ- CCTTGCACATGCCGGAG -3ˊ |
|  | R: 5ˊ- GCACAGAGCCTCGCCTT -3ˊ |
| METTL1 | F: 5ˊ- GGCAACGTGCTCACTCCAA -3ˊ |
|  | R: 5ˊ- CACAGCCTATGTCTGCAAACT -3ˊ |
| SMAD1 | F: 5ˊ- AGAGACTTCTTGGGTGGAAACA -3ˊ |
|  | R: 5ˊ- ATGGTGACACAGTTACTCGGT -3ˊ |
| SMAD2 | F: 5ˊ- CGTCCATCTTGCCATTCACG -3ˊ |
|  | R: 5ˊ- CTCAAGCTCATCTAATCGTCCTG -3ˊ |
| FGF2 | F: 5ˊ- AGAAGAGCGACCCTCACATCA -3ˊ |
|  | R: 5ˊ- CACAGCCTATGTCTGCAAACT -3ˊ |
| BMP4 | F: 5ˊ- ATGATTCCTGGTAACCGAATGC -3ˊ |
|  | R: 5ˊ- CCCCGTCTCAGGTATCAAACT -3ˊ |
| WNT2 | F: 5ˊ- CCGAGGTCAACTCTTCATGGT -3ˊ |
|  | R: 5ˊ- CACAGCCTATGTCTGCAAACT -3ˊ |
| OTX2 | F:5ˊ- CAAAGTGAGACCTGCCAAAAAGA -3ˊ |
|  | R: 5ˊ- TGGACAAGGGATCTGACAGTG -3ˊ |
| PAX6 | F: 5ˊ- TGGGCAGGTATTACGAGACTG -3ˊ |
|  | R: 5ˊ- ACTCCCGCTTATACTGGGCTA -3ˊ |
| SOX1 | F: 5ˊ- AGAAGAGCGACCCTCACATCA -3ˊ |
|  | R: 5ˊ- GCGGGCAAGTACATGCTGA -3ˊ |
| MEIS1 | F: 5ˊ- GCGGGCAAGTACATGCTGA -3ˊ |
|  | R: 5ˊ- GGGTACTGATGCGAGTGCAG -3ˊ |
| TBX6 | F: 5ˊ- CATCCACGAGAATTGTACCCG -3ˊ |
|  | R: 5ˊ- AGCAATCCAGTTTAGGGGTGT -3ˊ |
| MEOX1 | F: 5ˊ- GGGTACTGATGCGAGTGCAG -3ˊ |
|  | R: 5ˊ- GGTCCCCATTTCCTTGGAACC -3ˊ |
| PECAM1 | F: 5ˊ- AGCAATCCAGTTTAGGGGTGT -3ˊ |
|  | R: 5ˊ- TGTAAAACAGCACGTCATCCTT -3ˊ |
| NKX2-5 | F: 5ˊ- CCAAGGACCCTAGAGCCGAA -3ˊ |
|  | R: 5ˊ- GGTCCCCATTTCCTTGGAACC -3ˊ |
| ISL1 | F: 5ˊ- GGGTACTGATGCGAGTGCAG -3ˊ |
|  | R: 5ˊ- CACACAGCGGAAACACTCGAT -3ˊ |
| GATA4 | F: 5ˊ- CGACACCCCAATCTCGATATG -3ˊ |
|  | R: 5ˊ- GTTGCACAGATAGTGACCCGT -3ˊ |
| SOX17 | F: 5ˊ- GTGGACCGCACGGAATTTG -3ˊ |
|  | R: 5ˊ- GGTCCCCATTTCCTTGGAACC -3ˊ |
| FOXA2 | F: 5ˊ- GGAGCAGCTACTATGCAGAGC -3ˊ |
|  | R: 5ˊ- CGTGTTCATGCCGTTCATCC -3ˊ |
| OCT4 | F: 5ˊ- CTGGGTTGATCCTCGGACCT -3ˊ |
|  | R: 5ˊ- CCATCGGAGTTGCTCTCCA -3ˊ |
| SOX2 | F: 5ˊ- GCCGAGTGGAAACTTTTGTCG -3ˊ |
|  | R: 5ˊ- GGCAGCGTGTACTTATCCTTCT -3ˊ |
| NANOG | F: 5ˊ- TTTGTGGGCCTGAAGAAAACT -3ˊ |
|  | R: 5ˊ- AGGGCTGTCCTGAATAAGCAG -3ˊ |
| CyclinD1 | F: 5ˊ- GCTGCGAAGTGGAAACCATC -3ˊ |
|  | R: 5ˊ- CCTCCTTCTGCACACATTTGAA -3ˊ |
| CyclinD2 | F: 5ˊ- ACCTTCCGCAGTGCTCCTA -3ˊ |
|  | R: 5ˊ- CCCAGCCAAGAAACGGTCC-3ˊ |
| CDK2 | F: 5ˊ- GTACCTCCCCTGGATGAAGAT -3ˊ |
|  | R: 5ˊ- CGAAATCCGCTTGTTAGGGTC -3ˊ |

**Table S5.** Components of polysome cell extraction buffer

| Ingredients | Concentration |
| --- | --- |
| MOPS | 50 mM |
| MgCl2 | 15  mM |
| NaCl | 150  mM |
| cycloheximide | 100  μg/ml |
| Triton X-100 | 0.5% |
| heparin | 1  mg/ml |
| RNaseOUT | 200  U |
| PMSF | 2  mM |
| benzamine | 1  μM |
